# Multicellular force coordination constructs microchannel networks for barrier-free metastasis across extracellular matrix

**DOI:** 10.1126/sciadv.adz4291

**Published:** 2025-12-19

**Authors:** Huan Gao, Bo Cheng, Guorui Jin, Yulong Han, Qi Tian, Yan Zhou, Xiwen Zhao, Yan Liu, Chunyu Cao, Lizhe Zhu, Juan Zhang, Lin Wang, Binghe Xu, Hui Guo, Min Lin, Jin Yang, Feng Xu

**Affiliations:** ^1^ Phase I Clinical Trial Research Center, The First Affiliated Hospital of Xi’an Jiaotong University, Shaanxi 710061, P.R. China.; ^2^ The Key Laboratory of Biomedical Information Engineering of Ministry of Education, School of Life Science and Technology, Xi’an Jiaotong University, Xi’an 710049, P.R. China.; ^3^ Department of Cancer Center, The First Affiliated Hospital of Xi’an Jiaotong University, Shaanxi 710061, P.R. China.; ^4^ Bioinspired Engineering & Biomechanics Center (BEBC), Xi’an Jiaotong University, Shaanxi 710049, P.R. China.; ^5^ Department of Radiology, Hainan General Hospital (Hainan Affiliated Hospital of Hainan Medical University), NO. 19, Xiuhua St, Xiuying Dis, Haikou, Hainan 570311, P.R. China.; ^6^ State Key Laboratory of Mechanics and Control of Mechanical Structures, Nanjing University of Aeronautics and Astronautics, Nanjing 210016, P. R. China.; ^7^ Department of Radiology, The First Affiliated Hospital of Xi’an Jiaotong University, Shaanxi 710061, P.R. China.; ^8^ Department of Breast Surgery, The First Affiliated Hospital of Xi’an Jiaotong University, Shaanxi 710061, P.R. China.; ^9^ Engineering Research Center of Personalized Anti-aging Health Product Development and Transformation, Universities of Shaanxi Province, Xi’an International University, Shaanxi 710077, P.R. China.; ^10^ Department of Medical Oncology, National Cancer Center/National Clinical Research Center for Cancer/Cancer Hospital, Chinese Academy of Medical Sciences and Peking Union Medical College, Beijing 100021, P.R. China.; ^11^ Department of Medical Oncology, The Second Affiliated Hospital of Xi’an Jiaotong University, Xi’an 710004, P.R. China.

## Abstract

The extracellular matrix (ECM) acts as a primary physical barrier to cancer metastasis. While individual cancer cells can remodel ECM to create microchannel-like paths of least resistance, this cell-centric view overlooks the coordinated dynamics of multicellular communication. Here, we reveal that cancer cells collaboratively reprogram ECM to construct interconnected microchannel networks functioning as “superhighways” for barrier-free metastasis. Combining live-cell imaging, atomic force microscopy, and optical tweezers, we decode that the indispensable step in microchannel network construction is organized cross-convergence of adjacent channels. The convergence is precisely directed by mechanical bridges composed of aligned collagen bundles between adjacent channels, which transmit orientation cues to induce multicellular force coordination. Integrating single-cell sequencing and off-lattice agent-based model, we identify mechanically responsive leader cells enriched for integrin-RhoA/YAP signaling and matrix metalloproteinase 14, which sense bridge cues and initiate cross-convergence. Collectively, our findings unveil a self-organized metastatic network and its mechanobiological mechanisms, offering a previously unidentified framework and potential therapeutic insights for cancer metastasis.

## Teaser

Cancer cells engineer the ECM through mechanical teamwork, constructing superhighway networks that pave the way for metastasis.

## INTRODUCTION

Cancer metastasis contributes to the leading cause of cancer-related mortality (*1*), yet strategies for its effective treatment remain limited. The surrounding extracellular matrix (ECM) represents the first physical barrier that cancer cells must overcome to spread from the primary tumor and colonize distant organs (*2*). Intelligent cancer cells break through this barrier by constructing microchannels that provide paths of least resistance for escape (*3*–*5*). These structures can arise either through ECM degradation mediated by matrix metalloproteinases (MMPs; MMP-dependent mode) (*3*) or through force-driven ECM rearrangement (MMP-independent mode) (*4*, *6*). Existing evidence has primarily uncovered the local architecture of single microchannel and cancer cell activities inside (*7*–*10*). However, rather than operating in isolation, cancer cells interact continuously with the surrounding ECM (*2*) and communicate with neighboring cells across the matrix (*11*). The biophysical properties and biological significance of the metastatic microchannel clusters, particularly considering the inevitable multicellular communication, remain largely unexplored.

Intercellular communication among cancer cells, mediated by biochemical and physical signals, orchestrates their collective behaviors (*11*, *12*). Beyond well-known biochemical cues, ECM plays a unique role in generating and transmitting mechanical cues [e.g., ECM rigidity (*13*, *14*), fluid viscosity (*15*), and mechanical force (*16*)]. Cancer cells respond to these mechanical stimuli through mechanosensors such as integrins (*17*), which regulate the actin cytoskeleton dynamics and activate downstream pathways (*17*). In certain situations, the ECM functions as a medium that transmits mechanical cues between neighboring cells, a process termed mechanical communication (*18*). This is more than simply physical coupling because mechanical inputs are converted into biochemical signals and transduced into intracellular responses that coordinate collective behaviors (*18*). Cell-ECM-cell mechanical communication is implicated in acinar-to-invasive transitions (*19*) and mediates myofibroblast-fibroblast coordination at fibrotic borders, promoting fibroblast focus growth and fibrosis progression (*20*). Despite these advances, it remains unknown how such coordination influences multicellular metastasis within microchannels.

To address this, we combined long-term live imaging, traction force microscopy (TFM), atomic force microscopy (AFM), optical tweezers, single-cell RNA sequencing (scRNA-seq), and theoretical modeling to uncover how multicellular communication shapes microchannel architecture and to decode the underlying mechanisms. We initially unveiled an unprecedented microchannel network in both a multicellular three-dimensional (3D) in vitro system and in vivo breast cancer tissues, which paves the way for cancer metastasis. We delineated that mechanical bridges, composed of remodeled fiber bundles, couple neighboring microchannels, build a platform for cell-cell mechanical communication, and direct microchannel cross-convergence into a connected microchannel network. scRNA-seq analysis further identified leader cells enriched in integrin-Ras homolog family member A (RhoA)/Yes-associated protein (YAP) signaling as predominant mechanotransduction initiators. Together, our findings reveal a self-organized “expressway” microchannel driven by coordinated efforts of cancer cells, offering insights into the physical regulation of metastasis and potential strategies for therapeutic intervention.

## RESULTS

### Numerous microchannel-like invasion strands emerge as breast cancer cells infiltrate the surrounding ECM

**Fig. 1.**
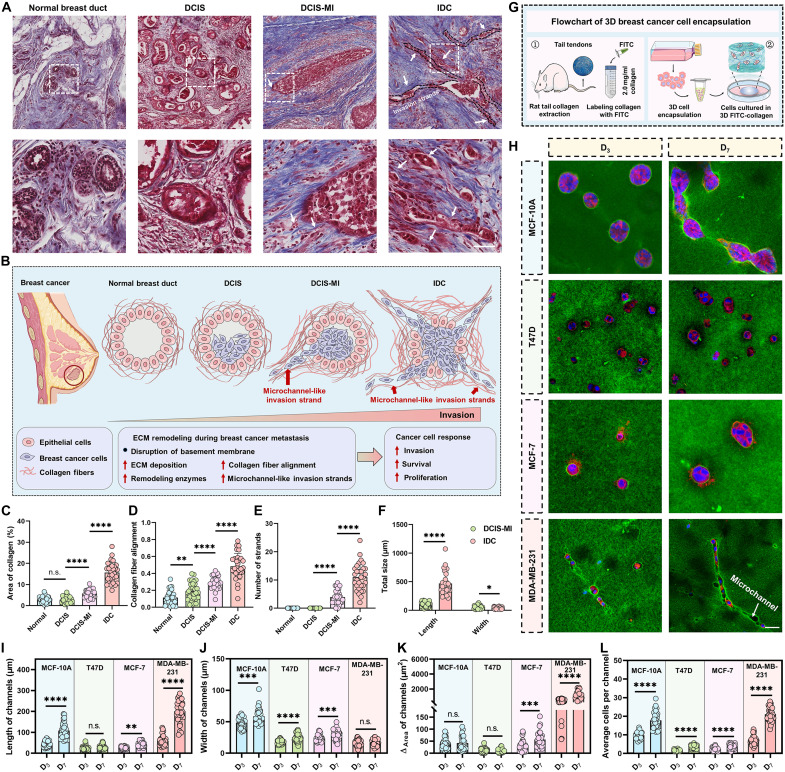
Numerous microchannel-like invasion strands emerge as breast cancer cells infiltrate the surrounding ECM. (**A**) Masson’s staining images of patients with breast cancer during breast cancer development. ECM was stained light blue. The white dashed boxes and the amplified images display the dynamic breast duct during breast cancer development. Arrowheads point to the microchannel-like invasion strands. Images are representative of three normal breast tissues and 60 samples from patients with breast cancer. (**B**) Schematic of ECM remodeling during progression, from normal duct to DCIS, DCIS-MI, and IDC, illustrating collagen deposition and alignment and emergence of microchannel-like strands. (**C**) Quantification of collagen area percentage across different breast cancer stages based on Masson’s staining. (**D**) Quantification of collagen fiber coherency across different breast cancer stages based on Masson’s staining. (**E**) The number of microchannel-like strands per square micrometers between different breast cancer stages. (**F**) Quantification of strand length and width in DCIS-MI versus IDC. (**G**) The workflow of rat tail collagen extraction, fluorescein isothiocyanate (FITC) labeling of collagen gels, and the construction of a 3D breast cancer cell culture model. (**H**) The dynamic process of microchannel formation within 3D matrix by breast epithelial cells (MCF-10A) and breast cancer cells (T47D, MCF-7, and MDA-MB-231). Cell plasma membrane (red), cell nucleus (blue), and FITC-collagen matrix (green). (**I**) Quantification of microchannel lengths generated by each cell type. (**J**) Width of microchannels generated by different cell types. (**K**) Measurement of the area of blank space within microchannels for each cell group. (**L**) Total cell numbers per microchannel in each kind of cells during culture. Data are presented as mean ± SD, and bubbles represent individual fields. *P < 0.05, **P < 0.01, ***P < 0.001, ****P < 0.0001, and n.s. (not significant). Scale bars, 50 μm.

To examine how tumor cells penetrate the surrounding ECM, we conducted Masson’s trichrome staining on 60 human breast cancer tissues and 3 paracancerous tissues, encompassing the progression from normal breast duct to invasive ductal carcinoma (IDC). The results demonstrate that at the preinvasive-stage ductal carcinoma in situ (DCIS), tumor cells remain confined by the basement membrane, which acts as a physical barrier. (Fig. 1, A and B). In DCIS with microinvasion (DCIS-MI), breast cancer cells break through the basement membrane and invade the surrounding ECM, with previously random collagen fibers become aligned ahead of the invading cells. At the IDC stage, the aligned collagen fibers assemble into microchannel-like tracks that encapsulate scattered or adherent breast cancer cells, forming stable multicellular invasion strands that infiltrate deeper into the surrounding ECM.

These histologic patterns illustrate ECM remodeling across stages (Fig. 1, A and B). Collagen area and fiber alignment increase with stage (Fig. 1, C and D), and the most aggressive subtype (HR^–^/HER2^–^) exhibits the greatest ECM remodeling, with higher collagen fiber alignment (fig. S1, A to C) and density (fig. S1, A and D). Consistently, the number of microchannel-like invasion strands increased (Fig. 1E). The widths of these strands decreased from 53 ± 32.6 μm in the DCIS-MI to 38.0 ± 16.4 μm in the IDC stage, while the lengths increased from 112.3 ± 40.0 to 436.4 ± 200.3 μm, indicating longitudinal extension of these tracks (Fig. 1F). These observations prompted the following question: How can breast cancer cells construct microchannel-like strands?

To address this, we encapsulated MCF-10A (nontumorigenic), T47D, MCF-7, and MDA-MB-231 cells, which differ in invasive potential, in fluorescein isothiocyanate (FITC)–labeled collagen hydrogels (2 mg/ml; Fig. 1, G and H, and fig. S1, E to G). Over 7 days, cancer cells remodeled collagen and generated such microchannels (Fig. 1H and fig. S1G). Notably, microchannels generated by MDA-MB-231 cells are significantly longer at both day 3 and day 7 culturing (D_3_: 60.0 ± 24.5 μm and D_7_: 206.3 ± 51.4 μm) (Fig. 1I) and slightly narrower (D_3_: 16.8 ± 4.9 μm and D_7_: 16.5 ± 5.0 μm) (Fig. 1J) compared with less invasive cell lines. To quantify resistance-free space, we calculated Δ_Area_ (microchannel area minus cell-occupied area). We observed that the microchannels generated by MCF-10A and T47D cells were largely filled with cells, leaving little Δ_Area_, whereas the microchannels generated by MCF-7 and especially MDA-MB-231 cells provided substantial Δ_Area_ (Fig. 1K). Δ_Area_ in MDA-MB-231 increased significantly from day 3 to day 7, along with an increase in cells per channel (Fig. 1L). These tissue and in vitro observations indicate that the aggressive breast cancer cells, particularly MDA-MB-231, remodel the ECM to construct elongated microchannels as they invade the surrounding matrix, providing low-resistance paths for multicellular invasion.

### Microchannel clusters are interconnected to form microchannel networks across the 3D collagen matrix

**Fig. 2.**
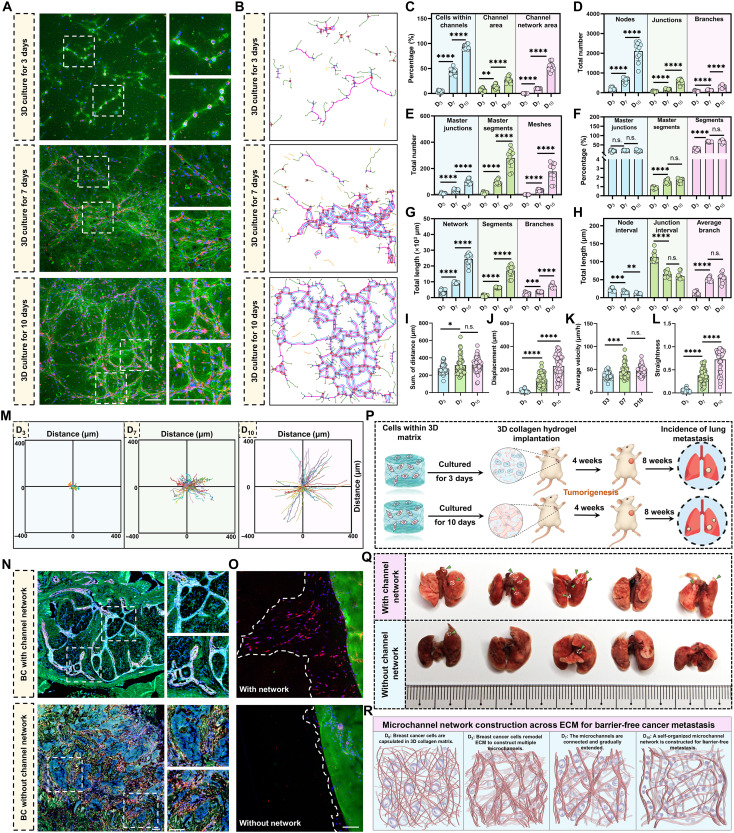
Microchannel clusters are interconnected to form microchannel networks across the 3D collagen matrix. (**A**) Confocal z-projections of MDA-MB-231 cultures in FITC-collagen at D_3_, D_7_, and D_10_. Collagen (green), plasma membrane (red), and nuclei (blue). Dashed boxes mark regions where isolated channels become interconnected. Enlarged views are shown at right. D_10_, day 10. (**B**) Reconstructed microchannel outlines from the fields in (A) for topology analysis in ImageJ. (**C**) Quantification of cells within microchannels, microchannel area, and microchannel network area over time. (**D**) Quantification of nodes, junctions, and branches in microchannel networks over time. (**E**) Analysis of master junctions, master segments, and mesh formation in microchannel networks over time. (**F**) Percentage of master junctions, master segments, and segments in microchannel networks over time. (**G**) Quantification of total microchannel length, segments, and branches over time. (**H**) Quantification of node and junction interval lengths, and average branch length over time. (**I** to **L**) Live-cell analysis of cancer cell migration, showing (I) sum of migration distances, (J) displacement, (K) average velocity, and (L) straightness, defined as displacement divided by the sum of migration distances (*D*/*S*). h, hour. (**M**) Representative trajectories of breast cancer MDA-MB-231 cells over time. (**N**) Immunofluorescence staining of microchannel networks in clinical breast cancer (BC) samples. (**O**) Invasion assay with or without a preformed network within the implanted scaffold. White dashed lines mark the invasive boundary. (**P**) Experimental design for subcutaneous implantation of collagen gels containing cells precultured without (D_3_) or with (D_10_) a network in nonobese diabetic (NOD)/severe combined immunodeficient (SCID) mice. (**Q**) Lung metastasis burden at endpoint with and without a microchannel network. (**R**) Schematic representation of the panoramic landscape of microchannel network formation across the 3D collagen matrix, illustrating the progression from isolated microchannels to fully interconnected networks. Data are presented as mean ± SD, and bubbles represent individual fields. *P < 0.05, **P < 0.01, ***P < 0.001, ****P < 0.0001, and n.s. (not significant). Scale bars, 100 μm.

Clinical tissues and 3D in vitro data confirmed the presence of metastatic microchannel clusters. Because MDA-MB-231 cells constructed the most stable microchannels, we select this cell line to explore the biophysical properties of microchannel clusters under multicellular communication. To tackle this, we lengthened the observation period to 10 days and expanded the analysis from single channels to multichannel clusters. 3D views show isolated microchannels at day 3, partial interconnections at day 7, and a junction-rich, mesh-containing microchannel network at day 10 (Fig. 2A and movies S1 to S3).

To analyze these structures, we generated 3D surface reconstructions (fig. S2A) and skeletonized the microchannels to map network topology over time (Fig. 2B, fig. S2B, and table S2), defining nodes, junctions, master junctions, branches, segments, and meshes. We then quantified the percentage of cells within microchannels, microchannel area, and the network area, all of which showed significant increase (Fig. 2C), along with increases in nodes, junctions, and branches (Fig. 2D). In addition, the results indicate a notable rise in master junctions, master segments, and meshes (Fig. 2E) and a corresponding increase in the percentage of these features (Fig. 2F). Specially, the total lengths of microchannel networks, segments, and branches also exhibited substantial growth (Fig. 2G). As the network formed, node-to-node and junction-to-junction interval decreased, whereas average branch length increased, indicating progressive convergence of channel clusters (Fig. 2H). Topology heatmaps revealed a maturation of microchannel architecture from day 3 to day 10, with stability, complexity, redundancy, robustness, and average degree all increased, indicating gradually construction of a highly connected, loop-rich architecture (fig. S2C).

We next assessed the motility consequences of the microchannel network. Notably, we observed that breast cancer cells migrate extremely more rapidly as the microchannel network developed (movies S4 and S5), with higher cumulative distance (Fig. 2I), displacement (Fig. 2J), average velocity (Fig. 2K), and straightness (Fig. 2L and fig. S2D). Real-time trajectory overlays illustrate the shift from short, winding paths to long, directed runs along network segments (Fig. 2M). Correlation analysis (fig. S2E) showed that straightness exhibited the strongest association with network architecture, with positive correlations to stability, redundancy, robustness, and average degree, followed by displacement. Average velocity displayed only modest associations, and total distance showed weak or nonsignificant correlations. Therefore, denser, loop-rich, and better-connected networks primarily enhance persistence and directionality, with only minor effects on speed, indicating improved migration efficiency. In clinical specimens, staining confirmed the existence of this microchannel network structures in vivo (Fig. 2N). To test whether networks facilitate escape into surrounding matrix, we embedded the periphery of the multimicrochannel system in blank collagen with or without a preformed network. Cultures containing a network showed more cells breaching the ECM barrier and infiltrating in an aligned direction, with a higher percentage of invasive cells (Fig. 2O and fig. S2, F and G). In vivo*,* implants with a preestablished network produced more metastatic lung nodules than implants without a network (Fig. 2, P and Q). These results indicate that isolated microchannels are gradually converge to form interconnected microchannel networks across the 3D matrix that support barrier-free metastasis (Fig. 2R).

### Remodeled collagen fiber bundles build “bridge” to direct microchannel cross-convergence for microchannel network construction

**Fig. 3.**
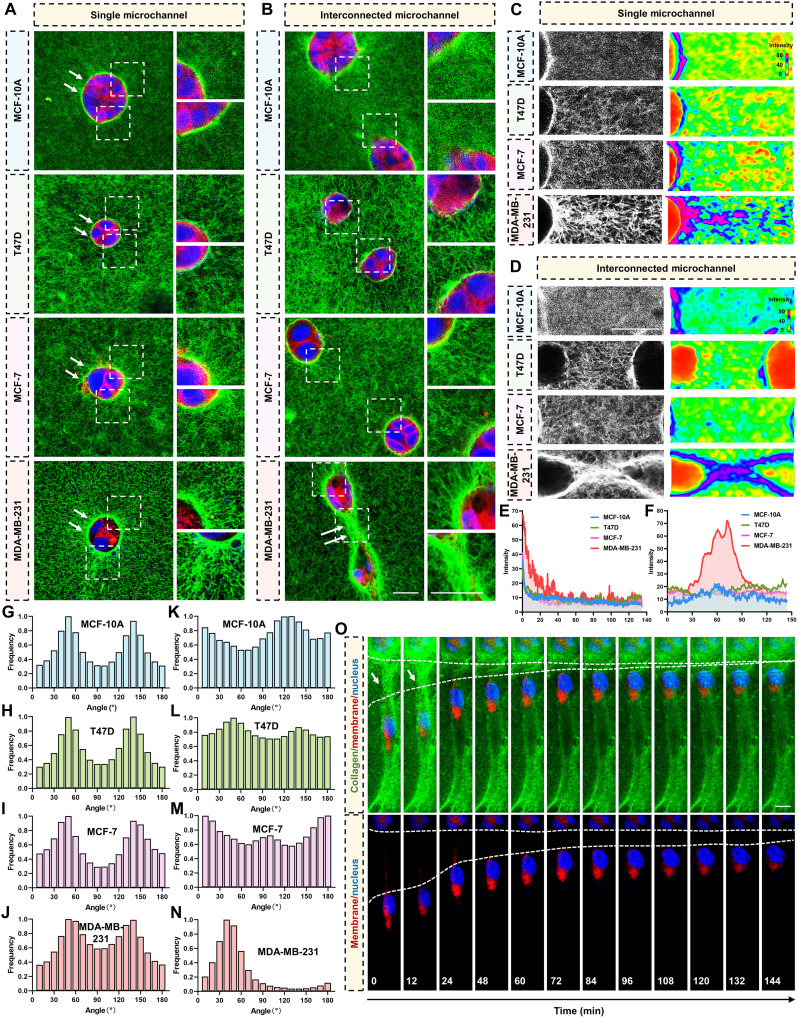
Remodeled collagen fiber bundles build bridge to direct microchannel cross-convergence for microchannel network construction. (**A** and **B**) Confocal images of (A) single microchannels and (B) paired/interconnected microchannels generated in 3D FITC-collagen by MCF-10A, T47D, MCF-7, and MDA-MB-231 cells. Collagen (green), plasma membrane (red), and nuclei (blue). Dashed boxes show magnified views. Arrowheads mark (A) channel walls and (B) collagen bridges linking two channels. (**C**) Collagen microarchitecture at single-channel walls. Left: Confocal reflectance (gray). Right: Matched fluorescence maps (ImageJ) of local collagen intensity. (**D**) Collagen microarchitecture at interconnected microchannels. Left: Confocal reflectance (gray). Right: Matched fluorescence maps (ImageJ) of local collagen intensity. (**E**) Quantification of the local fluorescence intensity of the microchannel walls in (C). (**F**) Quantification of the local fluorescence intensity of the microchannel connections in (D). (**G** to **J**) Orientation distributions of collagen around intact single microchannels for each cell line (OrientationJ). (**K** to **N**) Orientation distributions within the interconnected bridge region between microchannels for each cell line. Bridges formed by MDA-MB-231 cells exhibit the strongest parallel alignment, whereas interconnect are of other cells show broader angle distributions. (**O**) Time-lapse sequence of live MDA-MB-231 cells migrating along a collagen bridge toward the opposing channel, culminating in cross-convergence. Collagen (green), cell membrane (red), and nuclei (blue). Arrowheads mark the bridge and leading protrusions. Scale bars, 20 μm.

Given the role of the microchannel network in promoting cancer metastasis, we sought to investigate how such networks are constructed. We analyzed the collagen architecture around MCF-10A, T47D, MCF-7, and MDA-MB-231 cells, which differ in their ability to construct microchannels. Around single channels, all groups displayed radial collagen remodeling (Fig. 3A and fig. S3A). In paired-channel regions, only MDA-MB-231 cells exhibit a connecting structure composed of robust, thick collagen bundles spanning the gap between the two microchannels to form a bridge (Fig. 3B and fig. S3A). Notably, such a bridge is absent in other cell types.

To quantify ECM remodeling, we measured fluorescence intensity profile surrounding the single microchannel walls. Intensity maps revealed a perichannel decay extending ~40 μm from single-channel walls in MDA-MB-231 cells, compared with ~10 μm in the other cell lines (Fig. 3, C and E), indicating broader and stronger remodeling by MDA-MB-231 cells. In the interchannel region, the fluorescence intensity of MDA-MB-231 cells peaks at the bridge, whereas other cells maintain low and stable intensity values (Fig. 3, D and F). Fiber-orientation analysis showed radial collagen distributions around isolated channels in all groups (Fig. 3, G to J). In microchannel-connected areas of other cell types, the fiber arrangement is isotropic, distributing in all directions (Fig. 3, K to M), whereas bridges in MDA-MB-231 exhibited anisotropic, parallel alignment (Fig. 3N). Consistent with these architectural differences, MMP levels were highest in MDA-MB-231 among the tested cell types (fig. S3, B and C) and increased as the microchannel network developed (fig. S3, D and E). Together, these structural and biochemical features suggest the probability that the collagen bridges guide adjacent microchannels to approach and extend toward each other.

Next, to test guidance directly, we imaged the migration of MDA-MB-231 cells within bridged microchannels using time-lapse confocal fluorescence microscopy over a continuous period. Notably, cells in paired microchannels migrated toward and along the bridge, inducing cross-convergence of the two microchannels (Fig. 3O and movie S6). Collectively, these findings demonstrate that remodeled collagen fiber bridges serve as directional guides for cross-convergence of the bridged microchannels.

### Remodeled collagen bridges facilitate mechanical signal transmission to direct microchannel cross-convergence

**Fig. 4.**
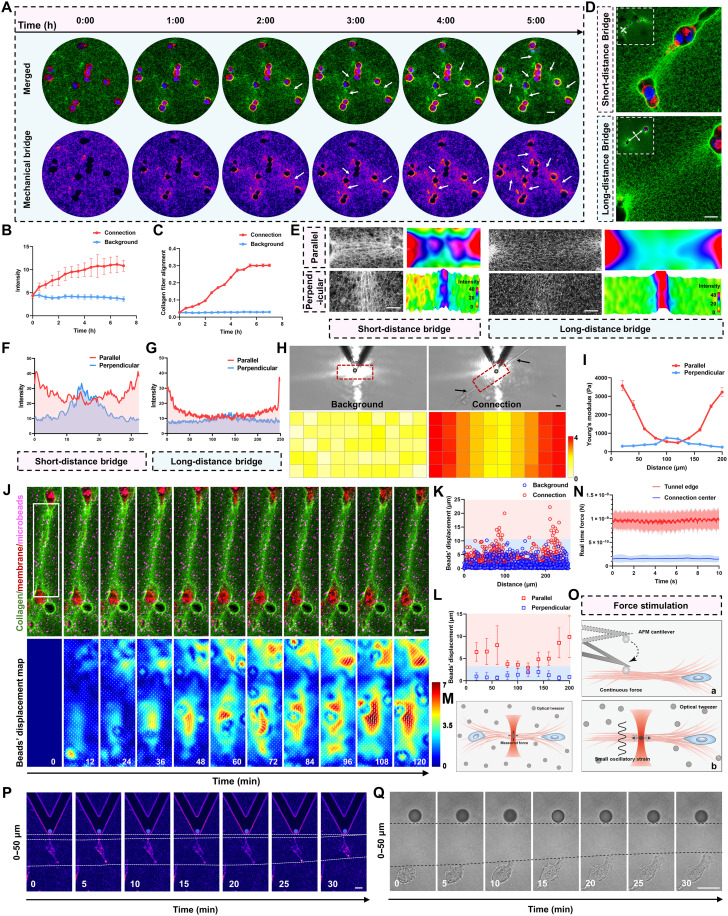
Remodeled collagen bridges facilitate mechanical signal transmission to direct microchannel cross-convergence. (**A**) Time-lapse confocal imaging shows breast cancer cells forming microchannel-microchannel connections within 5 hours. Arrowheads mark connections. Plasma membrane (red), cell nucleus (blue), and FITC-collagen (green). (**B** and **C**) Quantification over time of connection fluorescence intensity and fiber coherency from (A). (**D**) Typical images of microchannel pairs within 3D matrix with long-range (>121.4 μm) and short-range (<79.2 μm) distances. Plasma membrane (red), cell nucleus (blue), and FITC-collagen (green). Arrowheads marked the collagen paralleled and perpendicular to the connections. (**E**) Fluorescence maps of the arrowheads marked collagen fibers paralleled and perpendicular to the connections in (D); the top maps represent the paralleled direction, and the bottom maps represent the perpendicular direction. (**F** and **G**) Intensity distribution of collagen fibers along the parallel and perpendicular directions in short-distance (F) and long-distance (G) bridges. (**H**) Bright-field images and Young’s modulus distribution heatmap of AFM probing fields between the background and cell-cell connection area. The scale bar of the heatmap represents specific Young’s moduli in kilopascals. Arrowheads represent connected cells. (**I**) Distribution of the Young’s moduli of the collagen matrix paralleled and perpendicular to the cell connection. (**J**) Time-lapse images and beads’ displacement map in the white box of bridged microchannels. Plasma membrane (red), cell nucleus (blue), and FITC-collagen (green). (**K**) Beads’ displacement distribution across the connection regions compared to background areas shown in (J). (**L**) Beads’ displacement paralleled and perpendicular to the connection regions shown in (J). (**M** and **N**) Schematic of optical-tweezer tensile-force measurement and real-time force recorded along microchannel connections. (**O** to **Q**) Schematics of remote mechanical stimulation by AFM (A) and optical tweezers (B), and representative responses of MDA-MB-231 cell migration under 0- to 50-μm stimulation distances in 3D collagen. Data are presented as mean ± SD. Scale bars, 20 μm.

Given the role of the microchannel network in metastasis, we asked how adjacent microchannels become connected. Our observations indicate that remodeled collagen fibers form bridges that orient microchannel convergence and promote network assembly. These bridges create a continuous ECM linkage between paired microchannels, providing a plausible route for mechanical coupling without requiring direct cell-cell contact. To test this, we monitored bridge construction by time-lapse confocal microscopy. Notably, immediately after collagen gelation, MDA-MB-231 cells began remodeling the matrix into aligned bundles that bridged distantly cell clusters (Fig. 4A, movie S7, and fig. S3F). Within 5 hours postgelation, average fluorescence intensity and fiber alignment increased and remained stable thereafter (Fig. 4, B and C), indicating successfully establishment of stable bridges.

To uncover detailed information along the bridge, we characterized typically bridged microchannels with both short-distance and long-distance connections (Fig. 4D). On the basis of quantitative distribution analysis, bridges were classified into short (<79.2 μm), intermediate (79.2 to 121.4 μm), and long (>121.4 μm) categories. Notably, parallel to the microchannel bridge, the fluorescence maps exhibit symmetrically distributed decreasing gradients originating from the bridged microchannels, while perpendicular to the bridge, intensity peaked at the connection (Fig. 4E). Accordingly, the fluorescence intensity of collagen bundles along the connection shows a “U”-shaped fluctuation (Fig. 4F), whereas it produced an inverted U shape with a maximum at the midpoint (Fig. 4G). These spatial profiles indicate a collagen concentration gradient emanating from the leading edges of the bridged microchannels, consistent with directional mechanical cues along the bridged pair.

To assess the mechanical properties of theses bridges, we performed AFM to measure local stiffness in both the background matrix (fig. S4, A and B) and the bridge region (fig. S4, A, C, and D). Compared with background areas, the Young’s modulus was markedly elevated between the connected microchannels, with the highest stiffness (~3.4 ± 0.3 kPa) detected at the microchannel edges (Fig. 4H). These mechanical trends mirrored the fluorescence data, as Young’s moduli also showed a U-shaped pattern along the bridge and an inverted U shape across it (Fig. 4I). Supporting the presence of mechanical tension, microdissection of the collagen bundle under confocal imaging caused immediate separation of the cut edges (fig. S4E), indicating the existence of tensile forces across the bridge.

To visualize and quantify these forces, we embedded monodispersed fluorescent microspheres into the 3D collagen matrix and measured their displacement as a readout of local tension. Displacements were significantly elevated in the bridge region compared with the surrounding matrix, with maximal values near the leading edge of the microchannel bridge (Fig. 4, J to L). Using optical tweezers for direct force measurement, we found that the tensile force at the microchannel edge was approximately 0.9 nN (Fig. 4, M and N).

An underlying mechanism of these results is that cells within the microchannels sense tensile force signals for long-range mechanical communication, guiding the directional migration of cells within the bridged microchannel pairs. To test this, we applied compressive stress to collagen fibers along the cell axis using AFM-based single-cell stimulation to mimic the effect of a transmitted tensile signal (Fig. 4O-a). MDA-MB-231 cells responded to these inputs by migrating toward the source of stimulation when applied at distances of 0 to 50, 50 to 100, and 100 to 150 μm (Fig. 4P and fig. S4, F and H). In contrast, cells exhibited minimal displacement in the absence of stimulation (fig. S4, F and H). To more accurately mimic dynamic tensile cues, we further developed an optical tweezer–based platform in which optically trapped beads were sinusoidally displaced to stretch nearby collagen fibers (Fig. 4O-b). This stimulation again induced directional migration toward the tensile signal (Fig. 4Q and fig. S4, G and H), whereas no response was observed when beads remained stationary (fig. S4, G and H). Collectively, these findings show that remodeled collagen bridges both physically link adjacent microchannels and present mechanical cues that steer migration along the bridge, driving cross-convergence.

Because mechanical bridges guide microchannel convergence, we next asked what builds them. Tensile cues along bridges suggested a requirement for actomyosin contractility (*5*, *6*, *21*, *22*), so we inhibited cell contraction with blebbistatin. In parallel, prior work (*5*, *23*) and our Western blots identified MMP14 (fig. S3, B and C) as the predominant pericellular collagenase in 3D type I collagen in our system, so we blocked proteolysis by small interfering RNA (siRNA) knockdown of MMP14 (fig. S5, A and B). Both treatments reduced overall microchannel network construction (fig. S6, A and D to G). Only contractility inhibition eliminated the collagen bridges (fig. S6, B to D). Increasing contractility with narciclasine produced a modest rise in connectivity metrics such as junctions and master segments, with an upward trending for complexity, redundancy, robustness, and average degree (fig. S7, A to D). Together, these interventions indicate that actomyosin traction is required to form bridges, whereas MMP14 primarily supports channel remodeling and network growth.

### Single-cell transcriptomic analysis identifies a subpopulation enriched in integrin-RhoA/YAP signaling and MMP14

**Fig. 5.**
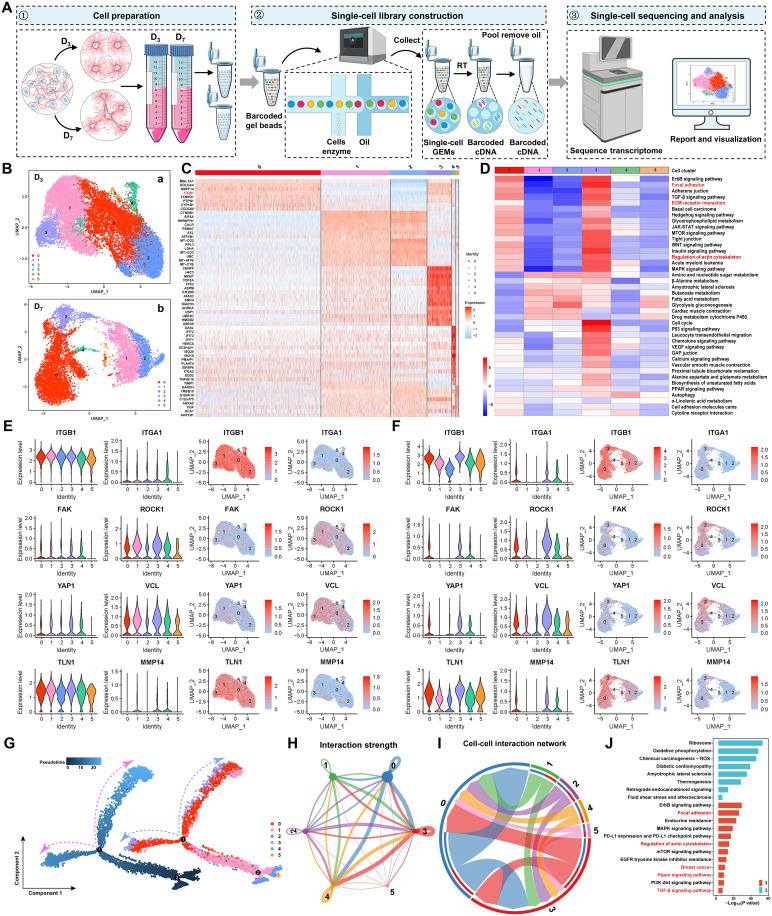
Single-cell transcriptomic analysis identifies a subpopulation enriched in integrin-RhoA/YAP signaling and MMP14. (**A**) Workflow of scRNA-seq for transcriptomic analysis of microchannel-forming cells. GEM, gel bead-in-emulsion. (**B**) UMAP visualization of MDA-MB-231 within 3D matrix at D_3_ (a) of 12,968 cells and D_7_ (b) of 7627 cells. (**C**) Heatmap of top 15 DEGs of the six clusters identified at D_7_. (**D**) Pathway activity of DEGs assessed by gene set variation analysis (GSVA) in each cell cluster at D_7_. (**E**) Violin plots and UMAP visualizations of marker genes from the six clusters at D_3_ (E) and D_7_ (**F**). (**G**) Pseudotemporal trajectories of all cancer cells (left) and of distinct clusters (right) at D_7_. Dots indicate single cells. (**H**) Interaction strength of the six cell clusters at D_7_. The thickness of the line represents the strength of the cell-cell interaction. (**I**) The circle diagram depicts the total number of ligand-receptor interactions between each subpopulation at each time point after amputation, where the connecting endpoints are sharp angles for ligands and arcs for receptors. (**J**) Kyoto Encyclopedia of Genes and Genomes (KEGG) pathway analysis from cluster 3 and cluster 2 (the lowest expression level of mechanotransduction genes and MMP14) at D_7_. The *P* values were determined using Fisher’s exact test.

Our current evidence showed that MDA-MB-231 cells build collagen bridges that transmit mechanical cues to direct microchannel convergence. To decode how cells sense and implement these cues, we performed scRNA-seq across network formation (Fig. 5A). Cells capsulated in collagen matrix are collected at D_3_ and D_7_, representing the early and intermediate stages of microchannel network development. After quality control (fig. S8, A to D) (*24*, *25*), a total of 12,968 cells in D_3_ and 7627 cells in D_7_ remained (fig. S8E). Uniform manifold approximation and projection (UMAP) analysis with Seurat (*25*) showed little substructure at D_3_ (resolution of 0.1), whereas D_7_ cells separated into six clusters (Fig. 5B-b). Increasing the D_3_ resolution to 0.2 also revealed six nascent clusters (Fig. 5B-a), indicating greater heterogeneity at D_7_. At D_7_, we identified 3167 differentially expressed genes (DEGs) [*P* < 0.05 and log fold change (logFC) > 0.25], with clusters 0 and 3 expressing high levels of integrin beta-1 (ITGB1) (Fig. 5C and table S3). Notably, cluster 3 exhibited the highest number of DEGs and was enriched in pathways related to focal adhesion, ECM-receptor interaction, and regulation of the actin cytoskeleton (Fig. 5D). In contrast, the D_3_ analysis revealed fewer DEGs (table S4) and weaker enrichment in ECM-related pathways (fig. S8, F and G).

Integrins such as ITGB1 and ITGA1 are key collagen receptors that mediate mechanotransduction via RhoA signaling, with downstream effectors focal adhesion kinase (FAK), ROCK1, YAP1, vinculin (VCL), and TLN1 (*26*–*28*). Comparative expression analysis of these markers showed similar expression levels across clusters at D_3_ (Fig. 5E), whereas at D_7_, higher expression levels of these markers were observed in clusters 0, 3, and 4 compared to clusters 1, 2, and 5 (Fig. 5F). Notably, cluster 3 showed the highest levels of Ki-67 (fig. S9A), as well as Slug, ZEB1, and Vimentin (fig. S9, M to P) that were associated with invasive cancer subtypes (*29*), indicating its invasive potential. Because microchannel convergence also requires ECM proteolysis, we assessed MMPs. Consistent with our Western blots (fig. S3, B and C), only MMP14 showed significant expression levels, especially in cluster 3 at D_7_, while other MMPs exhibited minimal expression and little variation across clusters (Fig. 5F and fig. S9, B to J). Moreover, mechanosensitive genes CAV1 and PIEZO1 were likewise enriched in cluster 3 (fig. S9, K and L).

Given these results, we hypothesized that the cell cluster with the activated mechanotransduction and high capacity of ECM degradation is primarily responsible for microchannel network formation. Pseudotime trajectory analysis positioned cluster 3 at a terminal state along the D_7_ trajectory (Fig. 5G). Moreover, ligand-receptor mapping revealed broad intercluster communication, with clusters 0 and 3 showing strong interaction strength (Fig. 5I) and the highest ligand output (Fig. 5H), indicating that cluster 3 likely coordinates network assembly. To further investigate the mechanistic pathways underlying mechanotransduction, we performed Kyoto Encyclopedia of Genes and Genomes (KEGG) enrichment analysis comparing cluster 3 with cluster 2 (the group with the lowest mechanotransduction markers and MMP14). Enriched terms included focal adhesion, actin cytoskeleton regulation, breast cancer, Hippo/YAP, and transforming growth factor–β (TGF-β) signaling (Fig. 5J), further supporting cluster 3 as the principal driver of microchannel network construction.

### Activated mechanotransduction and high MMP14 expression in leader cells highlight their dominant role in microchannel network formation

**Fig. 6.**
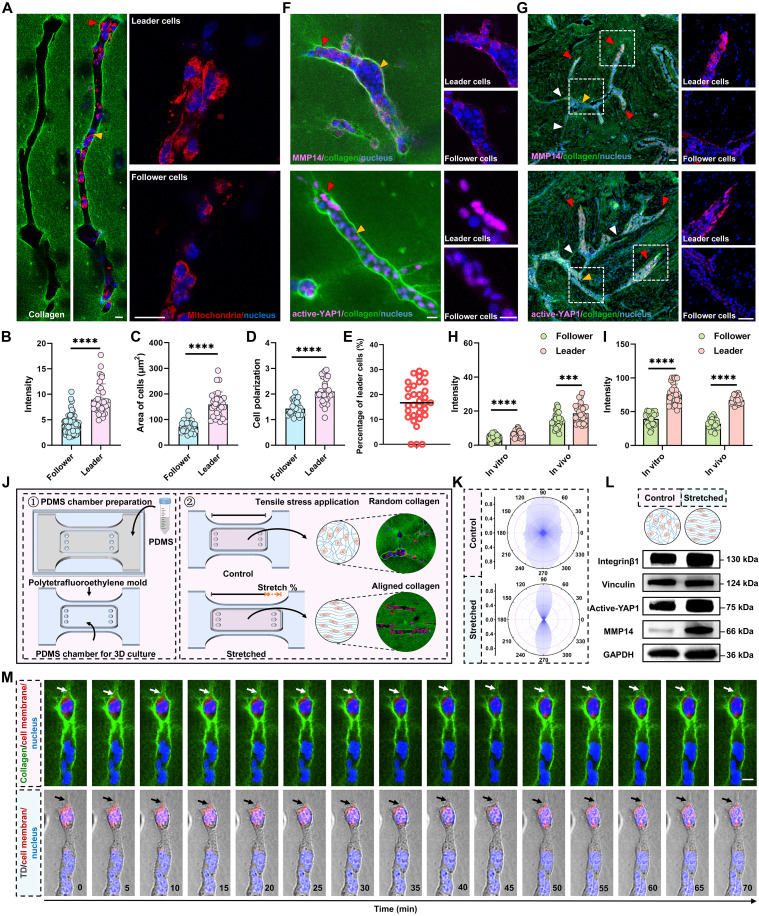
Activation of mechanotransduction and high MMP14 expression in leader cells highlight their dominant role in microchannel network formation. (**A**) Immunofluorescence staining shows leader cells (red arrowhead) and follower cells (yellow arrowhead) in the 3D collagen matrix. Leader cells exhibit increased mitochondrial activity (red) compared to follower cells. Cell nuclei are stained with 4′,6-diamidino-2-phenylindole (DAPI; blue), and collagen fibers are labeled with FITC (green). (**B** to **E**) Quantification of fluorescence intensity (B), cell area (C), cell polarization (D), and percentage of leader cells (E). (**F**) Image of MMP14 and active-YAP1 detection among breast cancer cells within microchannels in 3D multicellular system in vitro. Red arrowheads point to regions of intense MMP14 expression in leader cells, while yellow arrowheads point to follower cells. (**G**) Image of MMP14 and active-YAP1 detection among breast cancer tissues. Red and yellow arrowheads represent leader and follower cells, while the white arrowheads show the mechanical bridges. (**H** and **I**) Fluorescence quantification of MMP14 (H) and active YAP1 (I) in leader versus follower cells under in vitro and in vivo conditions. (**J**) Schematic of the polydimethylsiloxane (PDMS) chamber used for 3D culture, and the application of tensile stress to induce collagen alignment. The stretched group shows more organized collagen fibers than the control group with random fiber orientation. (**K**) Orientation distribution of microchannels in control and stretched groups. (**L**) Expression of mechanotransduction genes and MMP14 after mechanical stretching. (**M**) Time-lapse images of the mixed cells treated with Rho activator (pyrintegrin) or Rho inhibitor (verteporfin). TD, transmitted-light detector. Data are presented as mean ± SD, and bubbles represent individual fields. ***P < 0.001 and ****P < 0.0001. Scale bars, 20 μm.

Guided by the scRNA-seq result that a subpopulation is enriched for mechanotransduction genes and MMP14, we asked whether analogous “leader” cells exist in vitro. We labeled mitochondria with MitoTracker to estimate energetic state. Cells at the leading edge of single microchannels showed higher mitochondrial signal, larger mitochondrial size, and a more clustered organization than follower cells (Fig. 6, A and B, and fig. S10, A to C), indicating elevated metabolic activity. Morphologically, leaders were larger and more polarized (Fig. 6, C and D) and accounted for ~16.23% of cells within single channels (Fig. 6E). These observations indicated the presence of highly energy-consuming cells at the leading edge of single microchannels, highlighting the leading role of this subpopulation in microchannel formation. We next compared mechanotransduction and proteolysis markers in vitro and in clinical tissue. Leader cells at the microchannel front showed significantly higher MMP14 (Fig. 6, F and H), increased active-YAP expression, and an elevated active-YAP nuclear/cytoplasmic ratio compared with follower cells (Fig. 6, G and I, and fig. S10D). Moreover, the aligned collagen fibers between microchannels also validate the presence of mechanical bridges in breast cancer samples (Fig. 6G).

These results together indicate that leader cells at microchannel fronts exhibit greater ECM-remodeling capacity and heightened force responsiveness. Given that bridges carry tensile signals, external collagen alignment is expected to direct channel extension. We applied external stretch to generate an aligned collagen matrix for validation (Fig. 6J). Notably, microchannels formed by MDA-MB-231 cells oriented parallel to the fiber axis and the applied force direction (Fig. 6K and fig. S11, A and B) and showed greater length (fig. S11C), indicating that channel extension follows these mechanical cues. Immunofluorescence showed higher and more aligned MMP14 under stretch (fig. S11, D to F), and Western blots confirmed increased expression of integrin-RhoA/YAP pathway components and MMP14 (Fig. 6L and fig. S10E). These findings reinforce the molecular identity of leader cells and support a central role for mechanical cues in guiding microchannel extension.

Next, to test whether force-responsive cells lead channel extension, we activated RhoA with pyrintegrin and labeled those cells with CellMask (fig. S11, G and J). Labeled cells were mixed with unlabeled cells pretreated with verteporfin (fig. S11, H and I). After 7 days in 3D collagen, pyrintegrin-treated cells consistently occupied the front of advancing microchannels, whereas verteporfin-treated cells trailed behind (Fig. 6M and fig. S11K), indicating that cells with heightened mechanotransduction assume leader positions. Loss-of-function studies further supported the key role of integrin-RhoA/YAP activity and MMP14 in network assembly. Knocking down MMP14 by short hairpin RNA (shRNA) or inhibiting YAP signaling with verteporfin each inhibits network assembly, with fewer junctions, master segments, and meshes and lower topology scores (stability, complexity, redundancy, robustness, and average degree) (fig. S12, A to D). Combined inhibition [short hairpin MMP14 (shMMP14) plus verteporfin] produced the greatest effect, leaving short, isolated channels (fig. S12, A to D). Clinical and in vivo data reinforced the key role of MMP14. MMP14 staining was higher in tumors than adjacent tissue and in node-positive cases, and high MMP14 expression correlated with poorer survival (fig. S13, A to E). In the metastasis model, shRNA knockdown of MMP14 reduced lung colonization, and adding verteporfin further lowered metastatic burden (fig. S13, F to J). Collectively, these results show that cells with high integrin-RhoA/YAP activity and MMP14 function act as leaders that drive microchannel network construction, while coinhibiting either pathway significantly inhibits cancer metastasis.

### Off-lattice agent-based model captures the role of mechanical communication in driving microchannel network construction

**Fig. 7.**
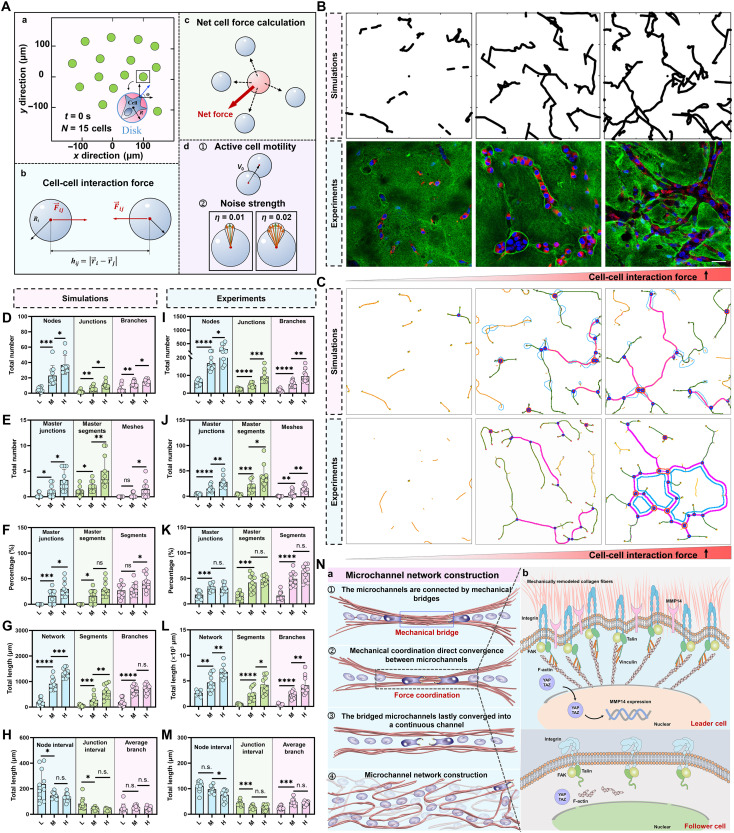
Off-lattice agent-based model captures the role of mechanical communication in driving microchannel network construction. (**A**) Schematic of the computational model simulating the interaction between cells during microchannel network formation. (**B**) Comparison of simulated microchannel networks and experimental data across different cell-cell interaction force. Top row: Simulated microchannel networks with varying cell-cell interaction force. Bottom row: Fluorescence images showing experimental microchannel network formation in a 3D collagen matrix with varying cell-cell interaction force. Contractility was modulated using different concentrations of blebbistatin: 20 μM (low interaction force), 5 μM (intermediate), and dimethyl sulfoxide (DMSO) control (high interaction force). (**C**) Complex contours of microchannels in simulations and 3D multicellular experiment systems under increasing cell-cell interaction force, with colors representing distinct microchannel structures. (**D** to **M**) Quantitative analysis comparing simulations and experimental results of microchannel network morphology under varying cell-cell interaction forces: low (L), middle (M), and high (H). [(D) and (I)] Quantification of nodes, junctions, and branches in simulated and experimental microchannel networks. [(E) and (J)] Analysis of master junctions, segments, and mesh formation in simulated and experimental microchannel networks. [(F) and (K)] Percentage of master junctions, master segments, and segments within the microchannel networks under varying cell-cell interaction forces. [(G) and (L)] Quantification of total microchannel length, segments, and branches within microchannel networks across different cell interaction force. [(H) and (M)] Quantification of node and junction interval lengths, and average branch length across different cell interaction force. (**N**) Schematic depicting the mechanistic insights from the study. (N-a) Mechanical bridges between microchannels direct microchannel cross-convergence, facilitating network formation. (N-b) Illustration of leader cells initiating microchannel network formation through MMP14 expression and integrin signaling. F-actin, filamentous actin. *P < 0.05, **P < 0.01, ***P < 0.001, ****P < 0.0001, and n.s. (not significant). Scale bars, 50 μm.

Mechanical bridges created by cell traction transmit tensile cues between neighboring microchannels. To examine how such mechanical communication drive network construction, we built an off-lattice agent-based model of migrating cells governed by local rules (Fig. 7A and Supplementary Methods). Individual cells are represented as disklike moving particles with a radius *R*, as illustrated in Fig. 7A-a. The state of each cell *i* is characterized by its position *r_i_(t)* and its direction of motion θ*_i_(t)* (Fig. 7A-a). The collective dynamics of migrating cells are governed by two primary factors: (a) cell-cell interaction forces and (b) active motion driven by protrusion force with directional noise. Cells interact via short-range interaction forces, where the elastic force *F_ij_* between any pair of cells *i* and *j* of radius *R* is given by the appropriate equation $F_{\mathrm{ij}}=F_{\text{base}}e^{d_{\text{dis}}/\alpha }$ (Fig. 7A-b). These interaction forces act along the unit vector *n_ij_*, pointing from the center of cell *j* to the center of cell *i*. The net force ${\vec{F}}_{i}$ on the *i*th cell is the vector sum of the elastic and adhesive forces exerted by the neighboring cells, ${\vec{F}}_{i}=\sum_{j\in \mathrm{NN}\left(i\right)}{\vec{F}}_{\mathrm{ij}}$ (Fig. 7A-c). The net contribution to the cell’s motility direction arises from two sources: the net interaction forces and noise in the direction of motion (Fig. 7A-d). Because channels form as cells move, the accumulated trajectories trace microchannel contours, which we skeletonized to quantify network topology.

To test how forces transmitted through collagen bridges shape network topology, we varied the interaction force in the model (Fig. 7B) and, in parallel, altered cell traction in MDA-MB-231 cells (Fig. 7B). Using the same methodology as in Fig. 2, we then applied the same analysis as in Fig. 2 to skeletonize channels and quantify network features (Fig. 7C). The analysis reveals a strong consistency between the simulation model and experimental data. Specifically, higher forces lead to an increase in the number of nodes, junctions, and branches, both in simulations and experiments (Fig. 7, D and I). The number of master junctions and segments also rises with increasing interaction forces, and mesh numbers increase as well, although to a lesser extent (Fig. 7, E and J). The percentage of master junctions becomes more dominant at higher forces, while the percentage of segments remains relatively stable, and the mesh percentage shows slight variation (Fig. 7, F and K). The total length of the network, segments, and branches expands notably, particularly in the transition from low to medium forces (Fig. 7, G and L). Besides these changes, the node interval length remains stable, while the junction intervals and average branch lengths increase slightly with higher forces, particularly in experiments, indicating that the network becomes more spaced out as interaction forces rise (Fig. 7, H and M).

To establish that leader cells are required for rapid network assembly, we encoded a leader subpopulation in the model and compared runs with or without leaders. With leaders, networks rapidly became junction rich and mesh bearing by day 10; without leaders, only short, scattered microchannels formed (fig. S14A and movies S8 and S9). Quantification confirmed the effect of leader cells in microchannel network construction (fig. S14, B and D). We also modeled cell-type heterogeneity by assigning measured parameter sets to each cell line. The simulations recapitulated the experimental hierarchy, with MCF-10A and T47D forming few, short tracks, MCF-7 showing intermediate connectivity, and MDA-MB-231 producing dense, highly connected networks (fig. S14E and movies S10 to S12). These matching trends validate the model and support a mechanism in which traction transmitted through collagen bridges promotes cross-convergence of microchannels.

## DISCUSSION

In this study, we delineate a self-organized microchannel network that functions as a dynamic expressway for barrier-free metastasis. By mapping its architecture and testing its biological impact, we show that aggressive breast cancer cells generate numerous microchannels and continue to remodel the ECM to build aligned collagen bridges that interconnect them. These bridges, driven by cell traction force, transmit mechanical signals and facilitate long-range mechanical communication between paired channels and direct cross-convergence to construct interconnected microchannel networks (Fig. 7N-a). ScRNA-seq further identifies a subpopulation at channel fronts enriched for integrin-RhoA/YAP signaling and MMP14 (Fig. 7N-b), positioning these cells to initiate mechanotransduction and drive network assembly. Together, these results decode the geometry and dynamics of microchannel networks in multicellular 3D systems, providing mechanistic evidence that force-driven collagen bridges transmit mechanical cues that direct cross-convergence to assemble interconnected networks. Conceptually, this framework highlights therapeutic opportunities that disrupt bridge formation or signal transmission, such as combined targeting of integrin-RhoA/YAP signaling and MMP14.

The ECM remodeling during breast cancer progression has been well studied, with increased ECM stiffness, fiber deposition, and alignment (*30*). We observed that aligned collagen bundles could be assembled into microchannel-like tracks, creating numerous multicellular invasion strands that infiltrate in all directions at the late stage of IDC. As multiple microchannels reappear in our multicellular 3D collagen matrix, accompanied with the previous evidence for broad cell-cell communication within ECM networks (*2**,* *31*), we then wonder the biophysical features of the metastatic microchannels, especially considering the inevitable multicellular communication. As we intend to trace the answer out, a panoramic view of microchannels, the microchannel network extending in all directions, was unmasked. Given that microchannel-like tracks provide migration paths with least resistance (*32*), we further demonstrated the metastasis superiority within the microchannel networks. Thus, the self-organized microchannel networks provide natural transportation routes for efficient cancer cell migration. In addition to using the “expressway network” to travel throughout ECM, cancer cells may also exploit the underlying path to efficiently convey biochemical cues (e.g., cytokines).

Analogous to neural or vascular networks, the interconnected microchannel network provides barrier-free paths that enable efficient long-distance communication and transport across the ECM. Consistent with this view, scRNA-seq with CellChat analysis revealed enrichment of cytokine secretion and signaling pathways (table S5), suggesting the potential role of the networked architecture to facilitate paracrine exchange along organized routes. Functionally, the network has a clear significance on migration. Correlation analysis reveal that denser, loop-rich, and better connected networks chiefly enhance straightness and directionality, thereby improving migration efficiency rather than merely increasing activity. Viewed through the lens of network theory, the geometry we observe parallels principles described in other biological networks, where connectivity and redundancy support reliable flow and resilience to local damage (*33*, *34*). By assembling bridges, junctions, and meshes, cancer cells may construct a robust metastatic microchannel network that sustains barrier-free dissemination even when individual paths are perturbed.

As directional guides, remodeled collagen fiber bundles between adjacent microchannels create a platform for coordinated cell behavior within the bridged pair. These bridges not only act as physical connectors but also relay mechanical signals over distance. Previous studies have established the role of collagen fibers in providing directional guidance for both single-cell migration (*20*) and collective cell movements (*35*). However, unlike the conventional view of unidirectional migration along preexisting collagen tracks, the ECM provides passive guidance cues to cells. Here, initially separate microchannels converge toward one another along traction-generated collagen bridges, demonstrating a bidirectional cross-convergence mechanism. This behavior suggests an active cell-ECM-cell communication process, whereby mechanical bridges facilitate reciprocal guidance and coordination between migrating fronts. This finding aligns with prior observations that traction forces can transiently reorganize collagen into tensile fiber bundles that coordinate collective migration (*36*). Extending this concept, our results show that mechanically remodeled collagen not only synchronizes movement but also provides a structural conduit for directional convergence of migrating cells. Notably, while earlier work indicated that such fiber bundles are transient and dissipate once cells meet (*36*), our study exhibited that the mechanical bridges are irreversible and robust, and when the leader cells move together for microchannel convergence, the microchannel bridge is remodeled into part of microchannel walls. This may be related to the fact that the previous study reasonably explained cell migration in normal tissues, such as tissue repair, while evidence from our demonstration provides better explanations for efficient cancer cell migration. Similarly, existing evidence showed that cell traction forces irreversibly drive the alignment of collagen bundles between single breast cancer cells (*37*) or epithelial mammary acini (*19*). Here, we move beyond instantaneous architecture to the long-term formation of microchannels and its consequences for invasion, corroborated by AFM, TFM/optical-tweezer force readouts, and single-cell transcriptomics that together support a bridge-mediated mechanotransduction mechanism. Notably, the bridge presented both tensile cues and a measurable stiffness gradient (~0.2 to 4 kPa), overlapping in magnitude with the self-generated gradients reported for epidermoid carcinoma cells (~0.5 to 2 kPa) that direct collective migration (*38*). Consistent with that report, our data further support a guidance role for the bridge-associated stiffness gradient during microchannel cross-convergence.

A large subset of these microchannels does not exactly descript the morphology of the microchannels (*23*, *39*, *40*). Instead of the microchannel edges consisting of collagen fiber fragments according to the images displayed, in our study, we observed that most of these microchannels show intact microchannel walls consisting of thick collagen bundles, which is closely in accordance with the clinical breast cancer samples. An appropriate explanation might be that most of the reported microchannels in literature are produced by cancer cells based on MMP degradation and a cell culture cycle of shorter than 3 days. In contrast, the breast cancer cells in our experiments were continuously cultured for 7 days. Rather than mechanical interaction through cell-cell junctions, we found that an intact microchannel structure here can keep the separated follower cells to maintain the same direction of invasion as the leader cells. Moreover, we observed that most follower cells do not extend microchannel width, while the leader cells prolong microchannel length until the “bridged” microchannel converges. Widening the microchannel only promotes larger tumor size, while prolonging microchannels promotes long-distance migration. We found that the leader cells are mostly present at the tips of both ends of the microchannel, or sometimes only at the end of one side, indicating that the extension of microchannels may be unidirectional.

Collective microchannel invasions are often led by cancer-associated fibroblasts or highly metastatic cancer cells, connected with follower cells by cell-cell adhesions (*41*). Prior work from Friedl and colleagues established that carcinoma cells migrate collectively along preexisting or cell-generated ECM tracks and can reuse and interconnect these paths to form multicellular “highways” in vivo and in 3D matrix (*8*, *42*–*44*). Building on this foundation, our study advances from track reuse to network assembly. We map a junction-rich, loop-containing microchannel network, quantify its topology over time, and identify the coordinated cross-convergence mechanism driven by mechanical communication. During microchannel invasion, follower cells communicate with leader cells through cell junction–based mechanical interaction, coordinately migrate with leader cells, and further expand the microchannel width (*39*, *45*). Consistently with the above demonstrations, we similarly identified that cells at the ends of microchannels exhibit more invasive ability with more active states of integrin-RhoA/YAP signaling cascades and MMP14 expression. Beside the outstanding capacity in mechanical cue response and ECM remodeling, the leader cells can also actively contract to produce tensile force to strengthen the mechanical bridge, due to the RhoA-dependent actin cytoskeleton dynamics (*17*). While no study has explored the cross-talk between these proteases and mechanotransduction, the scRNA-seq analysis identified potential regulatory molecules, such as tissue inhibitor of matrix metalloproteinase 1 (fig. S15) and ubiquitination-related genes (table S3). Beyond mechanosignaling, leaders are enriched for pathways such as ErbB receptor signaling pathway, endocrine resistance, mitogen-activated protein kinase (MAPK), and the programmed death-ligand 1 (PD-L1) checkpoint, indicating the potential predominant role of leader cells in metabolism and immune modulation.

In conclusion, by integrating clinical breast cancer tissues with 3D multicellular systems, we provide direct evidence that isolated microchannels self-organize into an interconnected network directed by mechanical communication along collagen bridges. These findings support a novel perspective on ECM remodeling, highlighting the role of cell-ECM-cell communication across the ECM matrix, and expand our understanding of the biological significance of microchannel formation. The resulting microchannel network provides expressway network for barrier-free metastasis, inspiring possibilities for these migration cells to convey diverse “cargos” more efficiently, such as biochemical cues. Mechanistically, we identify a predominant leader cell cluster with exceptional capabilities in mechanotransduction and ECM degradation, driven by the enrichment of integrin-RhoA/YAP signaling pathways and MMP14 expression. These findings open up therapeutic possibilities for cancer metastasis, such as targeting the disconnection of microchannel bridges or inhibiting leader cell mechanotransduction.

## MATERIALS AND METHODS

### Cell culture

The breast cancer cells, including MDA-MB-231 cells and ductal carcinoma MCF-7 and T47D cells, were obtained from the American Type Culture Collection. MDA-MB-231 cells were cultured in Leibovitz’s L-15 (KeyGEN) supplemented with 10% fetal bovine serum (FBS) (Thermo Fisher Scientific) and 1% penicillin-streptomycin (Hyclone). MCF-7 cells were maintained in Dulbecco’s modified Eagle’s medium (DMEM; Corning) and T47D cells in RPMI 1640 medium (Corning), both supplemented with 10% FBS, 1% penicillin-streptomycin, and 10 bovine pancreas insulin (μg/ml; Sigma-Aldrich). The nontumorigenic epithelial cell line MCF-10A cells were purchased from Beijing union medical college hospital cell resource-sharing platform. These cells were cultured in DMEM/F12 medium supplemented with 5% horse serum, human epidermal growth factor (20 ng/ml), insulin (10 μg/ml), hydrocortisone (0.5 μg/ml), and 1% penicillin-streptomycin. All cells were passaged using 0.25% trypsin (Hyclone) upon reaching 70% to 80% confluence. All cell lines were maintained at 37°C under a humidified atmosphere with 5% CO_2_.

### Clinical breast cancer sample collection

The collection of clinical samples was approved by the Institutional Review Board (IRB) in the Xi’an Jiaotong University Health Science Center (IRB no. 2021-602). Demographic and clinicopathological information, along with the paraffin-embedded tumor sections, were obtained from 60 patients with breast cancer treated with modified radical mastectomy from 2016 to 2019. Written informed consent was obtained from all participants before tissue collection and data use. For immunohistochemistry (IHC) staining of MMP14, we used commercial breast tissue microarrays (HBreD055cd01, Outdo Biotech, Shanghai, China) containing FFPE cores from six adjacent noncancerous tissues, 25 primary carcinomas, nine metastatic lesions, and 14 primaries with annotated nodal status (six are node-negative, and eight are node-positive).

### Masson’s trichrome staining

The clinical tissues were stained using a modified Masson’s trichrome staining (Solarbio, China) in accordance with the manufacturer’s instructions. Briefly, the 4-μm paraffin-embedded sections were deparaffinized, rehydrated, and incubated overnight at 37°C in Bouin’s solution (Solarbio, China) under a humidified atmosphere to prevent tissue drying. The slides were then gently washed with running tap water and stained with Mayer’s hematoxylin. After another wash, collagen content was stained after Ponceau S scarlet acid fuchsin, phosphomolybdic acid, and aniline blue. The slides were quickly dehydrated with 95% absolute alcohol and cleared in xylene. The degree of ECM remodeling was quantified by collagen deposition and fiber alignment (*46*), and we evaluated the length and width of the microchannel-like tracks following the previous quantification methods (*47*).

### Rat tail collagen extraction and fluorescent labeling of collagen hydrogels

Collagen fibers were extracted from rat tail tendons following the previous description (*48*, *49*). The harvested rat tail tendons were weighed and dissolved in a 0.1% acetic acid solution at a concentration of 100 g/ml for more than 48 hours at 4°C. Dissolved tendons were then centrifuged for 2 hours at 12,000 rpm at 4°C. The supernatant was collected and prefrozen at −80°C for 2 hours and then lyophilized for 72 hours. The dehydrated collagen was weighed and redissolved in 0.1% acetic acid at the stock concentration of 6 mg/ml. The obtained solution was preserved at 4°C until needed.

### Fluorescent labeling of collagen gels

The harvested type 1 collagen was labeled with FITC (Yeasen, China) as described previously (*50*, *51*). The lyophilized collagen was dissolved in 0.1 M sodium carbonate buffer (pH 9.0) at a concentration of 2.5 mg/ml for at least 48 hours. FITC powder was dissolved in dimethyl sulfoxide (DMSO) at a concentration of 1 mg/ml, which was then slowly supplemented into the collagen suspension at a ratio of 20 μl of FITC with 1 ml of collagen suspension. The mixed solution was allowed to react overnight before being quenched with ammonium chloride for 2 hours. Subsequently, final solution was dialyzed with 0.1% acetic acid for 48 hours. Then, the solution was lyophilized again and stocked at a concentration of 6 mg/ml. All the above steps should be protected from light to prevent the FITC label from quenching and keep at 4°C to prevent collagen inactivation.

### Type 1 collagen hydrogel preparation and 3D cell culture

FITC-labeled type I collagen pregels were prepared on ice by mixing collagen, phosphate buffered saline (PBS; 10×; Gibco), distilled water or cell suspension, and sodium hydroxide (NaOH; 0.2 M) to pH 7.4 to 8.0. The mixture was adjusted to a final collagen concentration of 2.0 mg/ml and a final cell density of 1 × 10^5^ cells/ml (Fig. 1G). To ensure true 3D encapsulation, cells were suspended in the neutralized pregel, and no surface seeding was performed. The pregel was dispensed into 15-mm glass-bottom confocal dishes (Nest, China) and polymerized for 45 min at 37°C in a humidified 5% CO_2_ incubator. Gels were cast with sufficient thickness (~1 mm) to position most cells away from top and bottom boundaries. Random volumetric encapsulation was verified by acquiring confocal z-stack, and representative 3D volume views are provided (fig. S1G). After gelation, complete growth medium was added, and cultures were maintained under standard conditions. To minimize proliferation-driven differences across groups, MDA-MB-231 cells were cultured in 2% FBS, which yielded minimal growth between D_3_ to D_7_ (10.56%) and D_7_ to D_10_ (10.91%) (fig. S2H).

### Immunofluorescence staining and confocal microscopy

Breast cancer cells capsulated in the collagen hydrogel were fixed with 4% paraformaldehyde for 10 to 20 min at room temperature, followed by cell permeabilizing with 0.1% Triton-X 100 (Sigma-Aldrich) for 10 min kept on ice. Then, the samples were blocked with 10% goat serum for 60 min. Solutions of primary antibody [rabbit monoclonal to MMP14 (1:500; Abcam) and rabbit monoclonal to active YAP1 (1:500; Abcam)] were introduced into the confocal dish and incubated overnight at 4°C. After the primary antibody incubation, the samples were slightly washed with PBS buffer and incubated with Alexa Fluor 647 goat anti-rabbit immunoglobulin G (IgG) (1:1000; Invitrogen) secondary antibody for 2 hours. Then, the secondary antibody–stained cell nuclei were stained with 4′,6-diamidino-2-phenylindole (DAPI; 1:1000; Sigma-Aldrich). Fluorescence and reflection mode of confocal microscopy were used to visualize images through ×10/0.40 numerical aperture (NA) and ×40/0.60 NA objectives (FV3000, Olympus). The fluorophores were excited by 405-, 488-, and 647-nm laser lines, and the detection windows were set according to the labels’ emission bands.

For tissue immunofluorescence staining, the paraffin-embedded tissue sections were baked at 60°C for 3 hours, deparaffinized in xylene, and rehydrated in a gradient of ethanol concentrations. Antigen retrieval was subsequently performed in sodium citrate buffer (pH = 9.0) using a microwave oven. Following permeabilization, the same procedures were used as for the 3D cell immunofluorescence staining.

For mitochondrial staining, to prepare a stock solution, the lyophilized MitoTracker Red product (Beyotime, China) was dissolved in DMSO to a final concentration of 200 μM. Then, the MitoTracker solution was diluted with growth medium to final working concentration (200 nM), followed by incubation with the final solution for 15 min under growth conditions. After incubation, the solution was carefully removed and washed three times with PBS. Then, the samples were fixed with prewarmed 4% formaldehyde, followed by nucleus staining. The images were captured by confocal microscopy. Fluorophores were excited by laser lines of 405 nm for DAPI, 488 nm for FITC-collagen, and 561 nm for MitoTracker, and the detection windows were set according to the labels’ emission bands. We further applied a zero-shot learning deconvolution/denoising network (ZS-DeconvNet) that self-trains on each image (no external training data) to suppress noise and enhance resolution.

### Real-time imaging

Live-cell time-lapse imaging was performed on a laser-scanning confocal microscope equipped with an environmental chamber maintained at 37°C, 5% CO_2_, and humidified air. MDA-MB-231 cells were labeled immediately before imaging with CellMask Orange plasma-membrane stain (1:1000; Invitrogen) and NucBlue Live ReadyProbes reagent (2 drops/ml; Invitrogen). After dye addition (per the manufacturer’s instructions), cultures were returned to the chamber, and excess dye was removed by a brief medium exchange. Time-lapse images were captured at regular intervals for a total duration to visualize dynamic cell activities. Acquisition intervals were task specific: cell migration: 15 min; microchannel cross-convergence: 12 min; collagen-bridge construction: 15 min; traction force mapping: 12 min; and leader-cell validation: 5 min. Total recording durations are provided in the corresponding figures.

### In vivo tumor experiments

All animal studies were conducted following the guidelines provided by the IRB in the Xi’an Jiaotong University Health Science Center (no. 2021-602). Four-week-old female nonobese diabetic (NOD)/severe combined immunodeficient (SCID) mice were obtained from the Laboratory Animal Center of Xi’an Jiaotong University and randomly assigned to groups with and without microchannel networks. MDA-MB-231 cells were embedded in type I collagen (2 mg/ml) as described for 3D culture. As showed in Fig. 2P, two cohorts were generated: a without-network cohort precultured for 3 days and a with-network cohort precultured for 10 days. To limit proliferation during preculture and preserve matrix remodeling, gels were maintained in 2% FBS. Growth profiling under these conditions showed minimal proliferation (D_3_ → D_7_: +10.56% and D_7_ → D_10_: +10.91%; fig. S2H).

Each mouse received ~1 × 10^6^ viable cells in a single collagen gel, implanted subcutaneously in the axillary region under isoflurane anesthesia. Because D_10_ gels spend longer in culture, we seeded them at 1.5× the initial density of D_3_ gels; before implantation, replicate gels from each batch were enzymatically dissociated (collagenase IV) and cell numbers were verified to ensure matched doses between cohorts (variation ≤20%). Tumor length (L) and width (W) were measured every 3 days. At 8 weeks posttumorigenesis, lung tissues were collected and processed for hematoxylin and eosin (H&E) staining, and the relative number of lung metastasis nodules was counted. Notably, cytoreductive surgery was performed on day 30 if tumor diameter approached ~1.5 cm, in accordance with ethical guidelines.

### Traction force microscopy

To measure cell-generated forces during bridge formation, FITC-labeled type I collagen (2 mg/ml) was mixed with 1-μm fluorescent microspheres (M197276, Aladdin; 5 ml) at a 1:10 (v/v) ratio and combined with MDA-MB-231 cells (1 × 10^5^ cells/ml). The mixture was cast into confocal dishes and cultured for 3 days to allow microchannel bridge formation. Before imaging, plasma membranes were stained with CellMask Orange (Invitrogen), and samples were maintained at 37°C with 5% CO_2_. Confocal images were acquired every 6 min for 2 hours, capturing FITC-collagen (488 nm), microspheres (405 nm), and cell membranes (561 nm). Microsphere displacement fields induced by cell traction were quantified using a custom MATLAB script previously validated for 3D matrix deformation analysis (*50*).

### AFM nanoindentation measurement

The stiffness of ECM was measured by NanoWizard BioScience AFM (Bruker) (fig. S4A), coupled with a microscope (Nikon) for positioning the AFM cantilever relative to the collagen matrix. To prepare spherical probes, a 12-μm green fluorescent microsphere (FL-PS-G-12, Dae, China) was glued to the tip of a silicon nitride cantilevers with nominal spring constants of 0.06 N/m (NP-O10 type D, Bruker). Before probing the collagen matrix, the cantilever was calibrated in a 1× PBS buffer at 37°C. “Contact mode force spectroscopy” was used in the JPK SPM control software with a *z*-speed of 2 μm/s and a setup *Z* length of 10 μm at a 1.8-nN set point. A total of 10 points was measured along (fig. S4C) or perpendicular to the bridged cells (fig. S4D). The maximum measurement duration of live cell within the collagen matrix was limited to 2 hours to minimize any negative impact of medium evaporation. AFM force-distance curves were analyzed by JPK Date Processing software (JPK BIOAFM) based on a contact-point–independent linear hertz mode (*51*, *52*), and Young’s moduli were determined with a Poisson ratio of 0.5.

### Tensile force measurement using optical tweezers

The NanoTracker 2 (Bruker) provides us an optical tweezer platform for real-time force measurements (*53*). To measure the tensile forces transmitted through collagen fibers across the 3D collagen matrix, we first mixed fluorescent microspheres of 2-μm diameter at 1:100 (v/v) (M197277, Aladdin; 5 ml) with cells at a concentration of 1 × 10^5^, capsulated the mixture in a collagen fiber matrix (2 mg/ml), and keep culturing until microchannel bridge structures were formed. Then, the laser beam (3 W and 1064 nm) was closely focused through a series of Keplerian beam expanders and a high-NA trapping objective (60×/1.4 oil; Nikon). Next, a high-resolution detection objective (60×/1.4 water; Nikon) was used for sample position detection. The fine-tune optical and the trap stiffness (0.4 pN/nm) calibration were performed with the same microspheres in distilled water. Then, microspheres located in the region of mechanical bridges are trapped, and real-time oscilloscope was applied for force measurement.

### AFM-applied force stimulation

AFM-based mechanical stimulation at the single-cell level was performed following established protocols referenced in recent studies by Du’s team (*20*). A NanoWizard BioScience AFM system (Bruker), coupled with a Nikon inverted microscope, enabled precise positioning of the cantilever tip relative to target cells. Silicon nitride cantilevers (NP-O10 type D, Bruker; nominal spring constant: 0.06 N/m) were functionalized with 12-μm-diameter green fluorescent microspheres (FL-PS-G-12, Dae, China) and calibrated in 1× PBS at 37°C before use. During stimulation, the microsphere-tipped cantilever was positioned adjacent to the cell boundary at a defined offset distance. A constant compressive force of 0.9 nN was applied for 30 min. Time-lapse confocal imaging was performed concurrently, capturing cell morphology and migration dynamics every 5 min throughout the stimulation period.

### Optical tweezer–applied force stimulation

Single-cell level of 3D tensile force stimulation was conducted by NanoTracker 2 (Bruker). At the beginning, MDA-MB-231 cells and fluorescent microspheres (FL-PS-G-12, Dae, China) of 12-μm diameter were embedded in 3D collagen matrix. Laser beam and samples were focused following the same procedure in force measurement. After fine-tune optical calibration, microspheres bridged with single cells were manipulated to conduct a continuous small oscillation along the mechanical bridge that could mimic the real-time movement of live cells. Time-lapse images of cell migration were captured for 30 min with a time interval of 5 min at different working distances.

### Single-cell preparation

To obtain the single-cell suspensions for single-cell sequencing analysis, MDA-MB-231 cells embedded in the 3D collagen matrix were isolated using collagenase IV (YEASEN, China). The 3D culture was incubated in a collagenase IV solution (0.5 mg/ml) for 15 min at 37°C. After the collagen gel dissolved, cells were centrifuged at 400*g* for 10 min. The pellet was washed three times with 1× PBS and resuspended in PBS with 1% FBS. The cell suspension was loaded into the MobiDrop microfluidic platform (Jiaxing, China) for droplet generation.

### Single-cell droplet generation and cell barcoding

Using the MobiDrop microfluidic platform, MobiNova-100, single cells, real-time reagents, and hydrogel beads were compartmentalized into nanoliter-scale droplets. These steps provide a unique barcode for each gel bead, and all cDNAs generated in a droplet share a common barcode. After LightCut treatment, the barcode primers attached to each gel beads in the droplets were released and compounded with the cell lysate and real-time reagent. The released primers contain (i) an Illumina read 1 sequence, (ii) a unique cell barcode, (iii) a 10-nt unique molecular identifier (UMI), and (iv) a poly–deoxythymidine sequence. Incubation droplets could produce barcoded, full-length cDNA from poly-adenylated mRNA.

### Single-cell cDNA amplification and library preparation

After cell barcoding, the droplets are broken, and the gel beads are removed through filtration. Solid-phase reversible immobilization (SPRI) beads (1.2×) are used to remove leftover biochemical reagents and primers in the aqueous phase. The recovered full-length and barcoded cDNA were then amplified to generate adequate mass for sequencing library preparation. Through a series of procedures including fragmentation, end-repair, A-tailing, adapter ligation, and SPRI size selection, Illumina P5 and P7 adaptor (including an i7 index) are added to the amplified cDNAs, and fragments with a suitable size are selected for sequencing.

### 3′-end single-cell sequencing

The MobiCube 3′ RNA-seq libraries were sequenced under a paired-end model on an Illumina instruments per the manufacturer’s instructions. Read 1 was used to sequence the cell barcode and the UMI, while Read 2 was used to sequence the cDNA fragment. The i7 index was used to distinguish samples.

### ScRNA-seq analysis

The raw data of single-cell sequencing (fastq format) are first analyzed by MobiVision v1.1. Reads are aligned to *Homo sapiens* reference GRCh38, and filtered cell-gene matrix was obtained. Further analysis was performed using the Seurat package in R software (v4.2.2) (*54*). Subsequently data quality assessing and filtering were performed, and those cells with more than 200 genes and less than 7000 genes and the percentage of mitochondrion genes less than 20% were filtered out. Then, the filtered expression matrix was used to identify cell subsets in each sample. For both sample analysis, we used log normalization of all matrix, through a size factor of 10,000 molecules per cell, followed by standardizing expression levels of all genes across the overall cells (*z*-score transformation).

To perform dimensionality reduction and visualization, the principal components analysis (PCA) was conducted. Because the structure of scRNA-seq data usually could not be captured by PCA analysis, the visualization algorithms were performed to create a 2D map for more comprehensively clustering of scRNA-seq data. To do this, the UMAP for dimension reduction (*55*) was conducted with a resolution of 0.1 at D_3_ and 0.2 in D_7_ analysis to reach the same clusters for comparing. The top 15 marker genes in each cluster of both groups were visualized by heatmaps. Because of the maker genes, the gene set variation analysis (GSVA) analysis was conducted by the msigdbr (*56*) and GSVA (*57*) package of R software to identify the enrichment signaling pathway among each cluster. To evaluate the expression level of mechanotransduction genes and MMP14, vinplot and featureplot algorithms were conducted. Next, to assess the time courses and spatial positions of all the cell clusters, the trajectory analysis was conducted by the Monocle 2 package of R software. Moreover, the CellChat (*58*) package was used for estimating the cell-cell interactions, including major signaling inputs and outputs, and the interaction strength between cell clusters. Besides, the KEGG (*59*) was further analyzed on the basis of DEGs between two specific cell clusters.

### Function inhibition and activation assays

For cell traction force inhibition, 20 μM (-)-Blebbistatin (TargetMol, China) was added to cultures to investigate the effect of inhibiting cell contraction on microchannel formation and interaction. Cell contraction activity was activated using omecamtiv mecarbil (CK-1827452, TargetMol, China) at the concentration of 0.5 μM, a selective myosin activator activating myocardial adenosine triphosphatase and improving energy utilization. For integrin-RhoA/YAP (HY-B0146, MCE) signaling inhibition, the verteporfin was used at a concentration of 200 nM. In contrast, the pyrintegrin (HY-13306, MCE) was used for integrin-RhoA/YAP signaling activation at a concentration of 0.5 μM. Down-regulation and up-regulation of integrin-RhoA/YAP–associated gene expression was verified by Western blot. To increase cell contractility, cultures were treated with narciclasine (HY-16563, MCE) at a concentration of 2 nM, which enhances RhoA/ROCK–myosin II signaling in nonmuscle cells (*60*).

### Transient down-regulation of MMP14

siRNA transfections were done with Lipofectamine 2000 reagent (Invitrogen) in an Opti-MEM serum-reduced medium (Gibco). Negative control and siRNA targeting MMP14 were purchased from GenePharma (China) with the following sequences: AACAGGCAAAGCTGATGCAGA. Down-regulation of gene expression was verified by Western blot.

### MMP14 knockdown by shRNA

To generate stable *MMP14*-knockdown cell lines, two shRNAs targeting human *MMP14* are cloned into lentiviral expression vectors by GeneChem (Shanghai, China). The target sequences were as follows: shMMP14#1, 5′-TGAGATCAAGGCCAATGTT-3′; shMMP14#2, 5′-GGATGGACACGGAGAATTT-3′. A scrambled sequence was used as a negative control (shNC). Lentivirus production and transduction into MDA-MB-231 cells were carried out using HiTransG P transfection reagent (GeneChem), followed by puromycin selection (2 μg/ml) for 2 weeks. Knockdown efficiency was validated by reverse transcription quantitative polymerase chain reaction (RT-qPCR) and Western blot analysis, and shMMP14#1 was selected for subsequent in vitro and in vivo experiments based on superior silencing efficiency (fig. S13, F to H).

### RNA isolation and RT-qPCR

Total RNA was extracted from cultured cells using TRIzol reagent (Invitrogen) according to the manufacturer’s protocol. RT was performed using the PrimeScript RT Master Mix (Takara, Japan) to synthesize cDNA. Real-time qPCR was carried out using a SYBR Premix Ex Taq kit (Takara) on a real-time PCR system to assess mRNA expression levels. The specific primers used for MMP14 and glyceraldehyde-3-phosphate dehydrogenase (GAPDH) were as follows: MMP14, 5′-CAGCAACTTTATGGGGGTGA-3′ (forward) and 5′-TGTCAAAGTTCCCGTCACAG-3′ (reverse); and for GAPDH, 5′-AGAAGGCTGGGGCTCATTTG-3′ (forward) and 5′-AGGGGCCATCCACAGTCTTC-3′ (reverse). Primers were synthesized by Sangon Biotech (Shanghai, China).

### SDS–polyacrylamide gel electrophoresis and Western blotting

Cells were digested by radioimmunoprecipitation assay buffer supplemented with protease and phosphatase inhibitors (TargetMol, China), and proteins were quantified using bicinchoninic acid assays (Beyotime, China). Proteins were separated by 10% SDS–polyacrylamide gel electrophoresis (Bio-Rad) and transferred to polyvinylidene difluoride membranes through a transfer apparatus (Bio-Rad). The membranes were then incubated with specific primary antibodies overnight at 4°C and secondary antibody for 2 hours at room temperature, followed by enhanced chemiluminescence developing solution incubation and fluorescence detection and the analysis of band intensities. Dilution of antibodies was used as follows: rabbit anti-MMP14 antibody (1:1000; Abcam), rabbit anti–integrin β1 antibody (1:1000; Abcam), rabbit anti-active YAP1 antibody (1:1000; Abcam), rabbit anti-VCL antibody (1:2000; Abcam), rabbit anti-GAPDH antibody (1:1000; Sigma-Aldrich), and goat anti-rabbit IgG antibody conjugated with horseradish peroxidase (HRP) (1:5000; Cell Signaling Technology). GAPDH was used as a loading control.

### Tensile force–induced collagen hydrogel preparation

First, a polydimethylsiloxane (PDMS) (DC-184, Dow Corning) chamber (cross-linking agent/monomer: 1/10) for cell culturing was cast using polytetrafluoroethylene mold. MDA-MB-231 cells were embedded in a concentration of mixture (2.0 mg/ml) of type 1 collagen hydrogel, which was prepared as in the previous step, and the mixture was subsequently added to the PDMS chamber. Then, after the mixture was gelatinized, a 10% stretching strain was applied to the PDMS chambers using a small horizontal stretching gripper (clamping range: 120 mm × 120 mm). No tensile force was applied to the control group. Both groups of cells were isolated at D_3_ culturing, and cellular proteins were collected for subsequent Western blot experiments to analyze mechanotransduction gene expression levels. Because the tensile force was performed directly on the collagen gel embedded in the PDMS chamber, rather than directly on the cells, the tensile force sensed by the cells is indeed transmitted through the collagen fibers, similarly to the tensile force between the leader cells transmitted along mechanical bridges. Thus, the experiment was expected to mimic (although perhaps not fully reproduce) the tensile force signal transmitted along the mechanical bridge, which enables us to determine the cellular response under force transmission through mechanical bridges, and the differences in molecular expression between leader and follower cells.

### Agent-based computational model

A detailed description of an agent-based computational model for microchannel network formation is given in the Supplementary Materials. Briefly, in this model, individual cells are represented as disklike particles that interact and move according to local rules. The model incorporates short-range cell-cell interaction forces and active motility driven by protrusive forces with directional noise. By simulating the dynamics of cell interactions and migration, the model aims to elucidate how cell interaction contributes to the formation of microchannel networks. Key parameters influencing cell interactions, friction with the ECM, and motility are varied to capture the processes underlying microchannel network formation.

### IHC and survival analysis

Immunohistochemical staining of MMP14 was performed following standard protocols. Briefly, paraffin-embedded tissue sections were baked at 60°C for 3 hours, deparaffinized in xylene, and rehydrated through a graded ethanol series. Antigen retrieval was carried out using sodium citrate buffer (pH 9.0) in a microwave oven. Endogenous peroxidase activity was quenched with 3% hydrogen peroxide for 15 min. After rinsing, sections were blocked with 5% bovine serum albumin for 30 min at room temperature to reduce nonspecific binding. Slides were then incubated overnight at 4°C with primary antibodies against MMP14 at 1:200. The following day, sections were incubated with species-specific HRP-conjugated secondary antibodies for 1 hour at room temperature. Signal detection was performed using diaminobenzidine substrate, followed by hematoxylin counterstaining. Slides were dehydrated through a graded ethanol series and cleared in xylene. For semiquantitative analysis, 10 random fields per tissue section were selected under a microscope, and the percentage of MMP14-positive cells was quantified to assess relative expression levels. Kaplan-Meier analysis was performed with the Kaplan-Meier plotter (https://kmplot.com/analysis/; RRID: SCR_024521) (*61*). We used the breast cancer RNA-seq module and queried MMP14. The endpoint was overall survival. The cohort was restricted to patients with triple negative breast cancer. We used the autoselected percentile with censor at threshold. Hazard ratios were estimated with Cox proportional hazards, and *P* values were obtained from the log-rank test.

### Tail vein injection lung metastasis model

To investigate the role of MMP14 in breast cancer lung metastasis, MDA-MB-231 cells were transduced with lentiviral vectors expressing an shRNA targeting MMP14 (shMMP14) or a scrambled control shRNA (shNC) (GeneChem, China). Knockdown efficiency was validated by RT-qPCR and Western blot analysis. For in vivo metastasis assays, 1 × 10^6^ viable cells were suspended in PBS and injected into the tail vein of 4-week-old female NOD/SCID mice. To evaluate the cooperative role of mechanotransduction signaling, mice were treated with verteporfin (100 mg/kg, administered intraperitoneally on days 3, 5, and 7 postinjection) with or without shMMP14. Mice were euthanized on day 28, and lungs were harvested for analysis. The number of metastatic nodules on the lung surface was quantified under a stereomicroscope and confirmed by H&) staining of paraffin-embedded lung sections.

### Image analysis

The percentage of collagen area in breast cancer tissues was determined using ImageJ software (*62*). Before analysis, we first calibrated the scale for each image in ImageJ using the embedded scale bar or metadata before making any measurements. The orientation of collagen fibers and cells encapsulated in collagen hydrogels was analyzed using ImageJ software with the orientationJ plugin. To measure the length and diameter of the microchannel, lines between the microchannel ends and both sides of the microchannels were drawn and measured by ImageJ. Results are reported as mean ± SEM and rounded to two decimal places. Notably, ImageJ computes distances with subpixel interpolation, so reported values can be finer than the pixel size. For immunofluorescence intensity quantification, regions of interest were manually defined on multichannel overlay images around individual cells, and relative signal intensities were extracted for each channel.

For microchannel network quantification, raw confocal image stacks were imported into Imaris (Bitplane), and the lumenized microchannel scaffolds were reconstructed as 3D surface objects using a machine learning–based segmentation approach. The reconstructed network masks were exported to ImageJ and converted into binary format. Morphological features were then extracted using the Angiogenesis Analyzer plugin (*63*), yielding parameters such as number of nodes, number of segments, number of branches, total network length, average segment length, and total mesh area. These outputs were exported to Excel and processed using a custom R pipeline (R 4.1.1) incorporating readxl, dplyr, stringr, janitor, and pheatmap packages. Network topology and function, including stability, complexity, tortuosity, redundancy, robustness, and average node degree, were computed for comparative analysis (*33*, *64*, *65*). For live-cell migration analysis, ImageJ was also used to identify and track the “*x*”, “*y*” and “*z*” coordinates of cell centroids. The recorded trajectories were processed using a custom Python script to reconstruct cell paths and compute migration parameters such as instantaneous velocity and straightness (*66*).

### Statistics analysis

All the cell culture experiments were conducted at least three times independently. Statistical analysis of data was performed using GraphPad Prism 8 software and presented as mean ± SEM. For comparisons between two groups, unpaired two-tailed Student’s *t* tests were used. Correlation analyses were also performed using GraphPad Prism. Pearson’s correlation coefficient (*r*) was used for normally distributed variables, while Spearman’s rank correlation was applied for nonnormally distributed data. Linear regression was used to assess relationships when appropriate, and correlation strength and *P* values were reported. Statistical significance was defined as *P < 0.05, **P < 0.01, ***P < 0.001, ****P < 0.0001, and n.s. (not significant).

## Supplementary Materials

### The PDF file includes:

Supplementary Methods Figs. S1 to S15 Tables S1, S2, and S6 Legends for tables S3 to S5 Legends for movies S1 to S12 References
