# Supplementary material for: Multicellular force coordination constructs microchannel networks for barrier-free metastasis across extracellular matrix
Source: Sci Adv. 2025 Dec 19;11(51):eadz4291. doi: 10.1126/sciadv.adz4291 (PMC12716403; doi:10.1126/sciadv.adz4291)
Supplement: Supplementary file 1 — Supplementary Methods Figs. S1 to S15 Tables S1, S2, and S6 Legends for tables S3 to S5 Legends for movies S1 to S12 References [file sciadv.adz4291_sm.pdf]

Science Advances
AAAS

Supplementary Materials for

Multicellular force coordination constructs microchannel networks for barrier-free metastasis across extracellular matrix

Huan Gao *et al.*

Corresponding author: Guorui Jin, jinguorui@xjtu.edu.cn; Binghe Xu, xubinghe@medmail.com.cn; Hui Guo, guohui@xjtufh.edu.cn; Min Lin, minlin@xjtu.edu.cn; Jin Yang, yangjin@xjtu.edu.cn; Feng Xu, fengxu@mail.xjtu.edu.cn

Sci. Adv. **11**, eadz4291 (2025)
DOI: 10.1126/sciadv.adz4291

The PDF file includes:

Supplementary Methods
Figs. S1 to S15
Tables S1, S2, and S6
Legends for tables S3 to S5
Legends for movies S1 to S12
References

Other Supplementary Material for this manuscript includes the following:

Tables S3 to S5
Movies S1 to S12

Supplementary Methods - Computational Model

Off-lattice Agent-based Computational Model for Microchannel Network Construction

To elucidate the role of the cell-cell interaction force in network formation, we study a dynamical system of particles with the ability to migrate and interact, governed by local rules. In this paper, we present an off-lattice agent-based computational model developed to study the growth and migration of cell collectives. Individual cells are represented as disk-like moving particles of radius R, as shown in Fig. A. In addition to its radius R, the state of each cell i is characterized by its position $r_i(t)$ and its direction of motion $\theta_i(t)$, as shown in Fig. A (inset). The collective dynamics of migrating cells is governed by two factors: (a) cell-cell interaction forces resulting from two-body interactions, (b) active motion driven by protrusion force with directional noise. The model implementation of these factors is described in detail below.

Individual cells interact with short-range interaction forces. The elastic force F_{ij} between any pair of cells i and j of radius R (Fig. B). This force is given by,

$$F_{ij} = F_{base} e^{d_{dis}/\alpha}$$

where F_{base} is the short-range attraction force, which increases with the distance between pairs of cells; d_{dis} is the distance between these two cells, α is the exponential constant. In our model, we assume that channels are formed during cell movement, so biological cells adhere to their immediate physical neighbors through cell adhesion molecules together in a channel.

The interaction forces act along the unit vector n_{ij}, which points from the center of cell j to the center of cell i. The net force (F_i) on the ith cell is the vectoral sum of the elastic and adhesive forces exerted on it by the neighbouring cells (Fig. C).

$$\vec{F}_i = \sum_{j \in NN(i)} \vec{F}_{ij}$$

In this context, the variable j is summed over the number of nearest neighbours, NN(i), of cell i. The nearest neighbors of cell i are all cells that satisfy the distance $h_{ij} < d_{\text{inter}}$. The net force due to cell-cell interactions is damped by an effective friction contribution, γ, resulting from a cell's interaction with the extracellular matrix (ECM), and we assumed that this resistance is related to MMP-mediated ECM degradation. The friction experienced by a cell is thus a quantity that is

independent of time but dependent on cell type. For example, the values of this quantity are greater in the MCF10A, T47D, and MCF-7 cell type due to lower MMP levels.

During cell migration, leader cells emerge from ordinary cells. At each simulation step, ordinary cells may transition into leader cells, and leader cells can revert to ordinary cells. Leader cells are characterized by lower effective friction (γ), stronger protrusion forces, and higher cell-cell interaction forces compared to ordinary cells. These properties enable leader cells to migrate faster and play a guiding role in the cell population's overall movement.

The complex dynamics of each cell within the collective is characterized by active motility. In the low Reynolds number limit, the equation of motion is fully described by the following update rules

$$r_i^x(t+\Delta t) = r_i^x(t) + v_0 \cos(\theta_i(t))\Delta t + \frac{F_i^x(t)}{\gamma_i}\Delta t$$

$$r_i^y(t+\Delta t) = r_i^y(t) + v_0 \cos(\theta_i(t))\Delta t + \frac{F_i^y(t)}{\gamma_i}\Delta t$$

$$\theta_i(t+\Delta t) = \theta_i(t) + \eta\varepsilon_i$$

The above equations describe the evolution of the x and y coordinates of a cell i, which is propelled by an active component with a speed v_0 in the direction $\theta_i(t)$ at a time driven by the net force on the cell due to its contacting neighbors. The third equation describes the orientation dynamics of a cell, where $\theta_i(t + \Delta t)$ is the direction in which the cell moves in the next time step. The net contribution to the direction of a cell's motility arises from two sources: the net interaction forces and noise in the direction of motion (**Fig. 7A-d**).

The simulations were initiated by generating 15 non-overlapping cells, which were randomly distributed in a square region within a 2D spatial domain of size 200 μm × 200 μm. For all future time steps, an open boundary condition was considered. Each cell was assigned an initial orientation of the active velocity, which was randomly distributed in the domain [0, 2π]. Fluctuations in the direction of a cell's motion are captured by a noise term, which is randomly distributed with uniform probability in the range [−π/6, π/6]. The strength of the fluctuations is denoted by η. In the present study, all parameters are fixed except the γ, which is varied from 0.1 to 10. The simulated cell migration is evolved for approximately 20,000 steps. The relevant

parameters are shown in the following **Table S1**. A fixed timestep of 0.1 seconds was employed. The particle coordinates were recorded and utilized to compute the dynamical observables pertinent to the present study.

Tables S1. Model Parameters

Parameters	Values
Time step (Δt)	0.1 s
Active cell speed (v_0)	0.5 μm/s
Critical radius of cell (R)	50 μm
Noise strengths (η)	0.01-0.2
The short-range attraction force F_{base}	500 pN
Resistance for cell migration λ	0.1 kg/(μm·s) for MB 231 and 10 kg/(μm·s) for MCF-7, 15 kg/(μm·s) for T47D, 20 kg/(μm·s) for MCF-10A

Supplementary Methods - Microchannel Network Analysis

Table S2. Definitions of Tunnel Network Geometry Features

Structure Features	Definition
Node	Points where one or more branches of the tunnel network meet.
Junction	Special types of nodes where two or more branches of the tunnel network intersect.
Branch	The linear or curvilinear segments connecting one node or junction to another.
Segment	Smaller divisions of the network, typically representing the sections between two nodes.
Master Junction	Key or primary junctions in the network where multiple connections (typically more than two branches) intersect.
Master Segment	The principal or primary segments connecting master junctions.
Mesh	The interconnected structure formed by multiple branches and junctions in the tunnel network.
Node and Junction Interval	The distances between successive nodes or junctions along a branch. These lengths are used to characterize the spacing and connectivity of the network.

Network metrics

Let n be the number of nodes, m the number of segments (edges), b the number of branches, A the analyzed area,

L_{avg} the average segment length, and L_{tot} the total segments length. We use a small constant $\varepsilon=10^{-6}$ to avoid division by zero where noted. All metrics were computed per image.

Average degree:

$$k = \frac{2m}{n}$$

Connectance (link density):

$$C = \frac{2m}{n(n-1)}$$

(Fraction of realized links among all possible undirected links.(*67*))

Redundancy:

$$Redundancy = \frac{m}{b+\varepsilon}$$

Branch density:

$$\rho_{\text{branch}} = \frac{b}{A}$$

Global efficiency quantifies how efficiently a network exchanges information:

$$E = \frac{1}{n(n-1)} \sum_{i \neq j} \frac{1}{d_{ij}}$$

Where $d_{i,j}$ is the shortest-path distance between nodes *i* and *j*(*68*).

Tortuosity. When all length terms are available, tortuosity are estimated as:

$$\tau_{\text{est}} = \frac{L_{\text{tot}}}{mL_{\text{avg}}}$$

If a required term is missing, we use a geometry-based fallback,

$$\tau_{\text{alt}} = \frac{L_{\text{avg}}}{\sqrt{A/m}}$$

and report $\tau = \text{coalesce}(\tau_{\text{est}}, \tau_{\text{alt}})$, i.e., the first non-missing value. (The coalesce operation returns the first available value in a sequence; tortuosity follows standard path-length/Euclidean concepts.(69))

Robustness.

Let the isolated fraction be

$$iso = \min(\frac{iso\_segments}{m}, \frac{iso\_branches}{m}, 1)$$

Where iso_segments is the count of orphaned segments (single-segment components not connected to the main network) and iso_branches is the count of orphaned branches (disconnected branch elements) reported by image-analysis pipeline; if these counts are unavailable, they are treated as 0. We then define a connectivity-based proxy:

$$Robustness = (1 - iso) \cdot k$$

Motivated by error/attack tolerance in complex networks(70).

Composite complexity. To enable comparability across heterogeneous units, we form a weighted z-score blend:

$$Complexity = 0.3z(k) + 0.3z(C) + 0.2z(\rho_{\text{branch}}) + 0.2z(Redundancy)$$

Where $z(x) = (x - \mu_x) / \sigma_x$ is the across-dataset standard score.

Composite stability:

$$Stability = 0.5z(Robustness) + 0.5z(Complexity)$$

(For visualization we also report 0-1 rescaled versions of the composites.)

Supplementary Figures

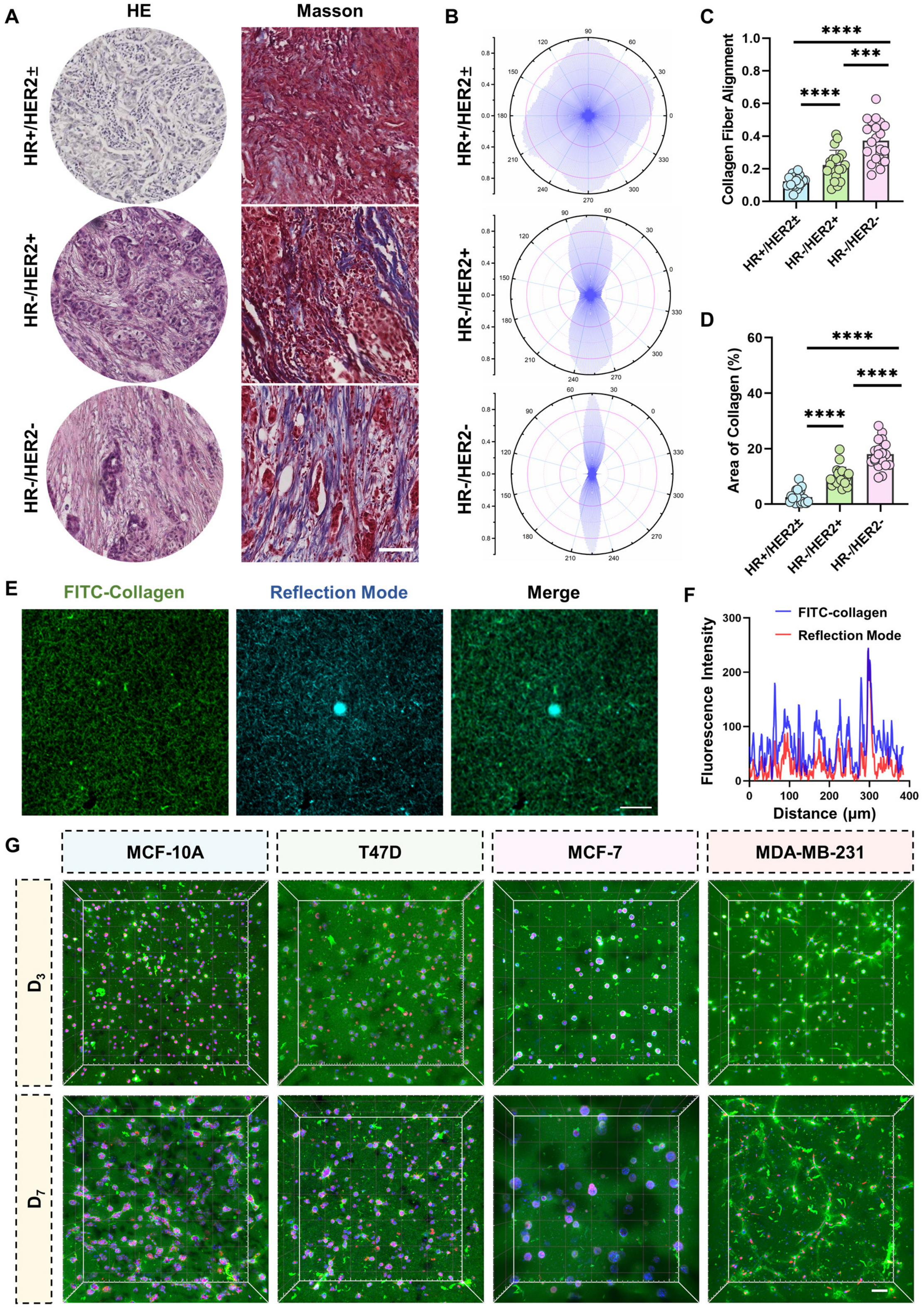

Fig. S1. ECM architecture differs across breast cancer subtypes and cell models. A. Representative H&E and Masson's trichrome images from patient tumors grouped by molecular subtype (HR+/HER2±, HR-/HER2+, HR-/HER2- HR, hormone receptor). B. Orientation distribution of collagen fibrils in different molecular subtypes of breast cancer. C. Quantification of collagen fiber alignment of different molecular subtypes based on Masson's staining. D. Quantification of percentage collagen area of different molecular subtypes based on Masson's staining. E. Representative images of FITC–labeled rat-tail type I collagen (fluorescence), confocal reflectance image of the same field, and merge, confirming that FITC signal reports collagen fibrils. F. Line-scan intensity profiles for FITC (green) and confocal reflectance (blue) along the indicated path in (E). G. 3D collagen cultures (2 mg/mL) of MCF-10A, T47D, MCF-7, and MDA-MB-231 at day 3 (D_3) and day 7 (D_7). Unpaired two-tailed t-test. ***P <0.001, ****P < 0.0001. Scale bar is 100 μm.

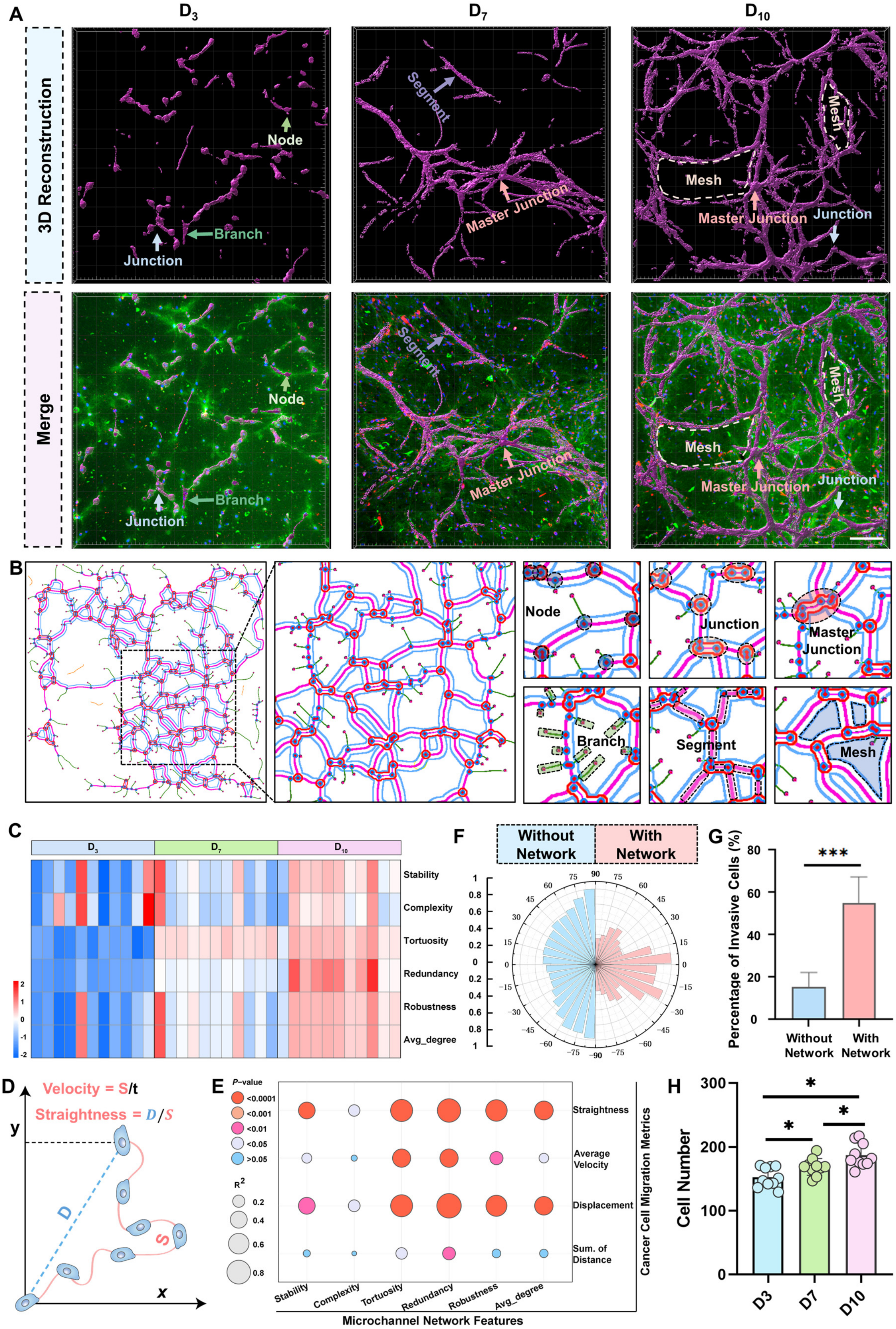

A
D3
D7
D10
3D Reconstruction
Merge
Node
Branch
Junction
Segment
Master Junction
Mesh
B
Node
Junction
Master Junction
Branch
Segment
Mesh
C
Stability
Complexity
Tortuosity
Redundancy
Robustness
Avg_degree
F
Without Network
With Network
G
Percentage of Invasive Cells (%)
***
Without Network
With Network
D
Velocity = S/t
Straightness = D/S
E
P-value
<0.0001
<0.001
<0.01
<0.05
>0.05
Straightness
Average Velocity
Displacement
Sum. of Distance
Cancer Cell Migration Metrics
Microchannel Network Features
H
Cell Number
D3
D7
D10

Fig. S2. Microchannel networks are constructed across the 3D matrix for barrier-free metastasis. A. 3D reconstructions (magenta) of microchannels generated by MDA-MB-231 cells in 3D FITC-collagen (2 mg/mL) at day 3 (D_3), day 7 (D_7), and day 10 (D_{10}). Bottom row, merges with FITC-collagen (green) and nuclear/membrane stains (blue/red). Representative network features (node, junction, master junction, branch, mesh) are annotated. B. Skeletonized maps of the same fields (left: full field; middle: zoom) and examples of key network features (right panels: node, junction, master junction, branch, segment, mesh). C. Heatmap of network topology over time (normalized values): stability, complexity, tortuosity, redundancy, robustness, and average degree. D. Definition of velocity (S/t) and straightness (D/S) during MDA-MB-231 migration. E. Correlation bubble plot linking network features to cell migration (sum of distance, displacement, average velocity, and straightness). Dot size reflects correlation magnitude; color encodes *P* value. F. Polar histograms of migration orientation without (left) and with (right) a pre-formed microchannel network. G. Percentage of invasive cells in cultures without *vs.* with a pre-formed network. H. The proliferation levels of cells at different among the D_3, D_7 and D_{10} culturing. Bubbles represent the total number of cells per individual field. Unpaired two-tailed t-test. *P <0.05, ***P < 0.001. Scale bar is 100 μm.

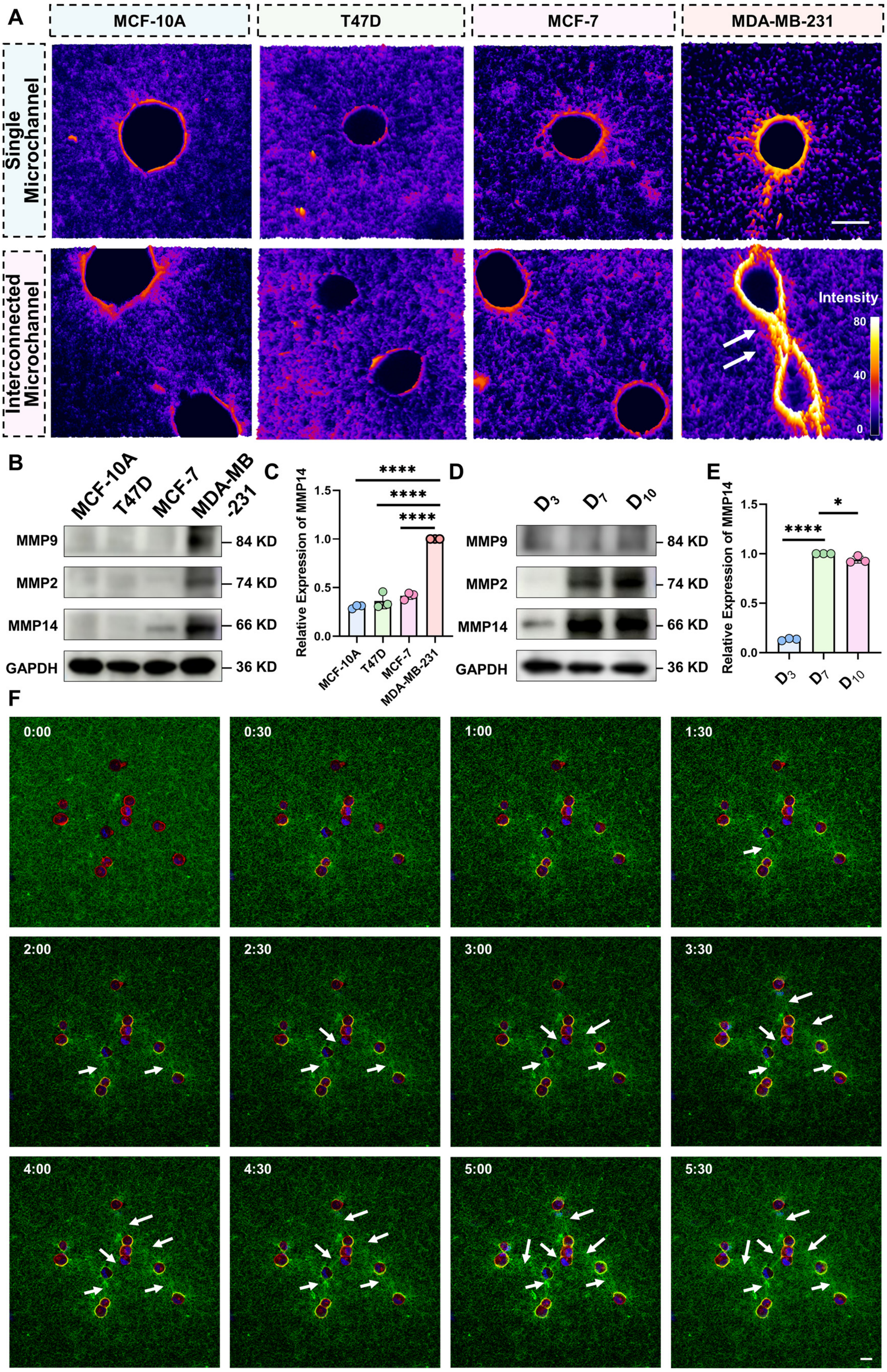
A
MCF-10A
T47D
MCF-7
MDA-MB-231
Single Microchannel
Interconnected Microchannel
Intensity
80
40
0
B
MCF-10A
T47D
MCF-7
MDA-MB -231
MMP9
MMP2
MMP14
GAPDH
84 KD
74 KD
66 KD
36 KD
C
Relative Expression of MMP14
****
D
D3
D7
D10
E
F
0:00
0:30
1:00
1:30
2:00
2:30
3:00
3:30
4:00
4:30
5:00
5:30

Fig. S3. Remodeled fiber bundles bridge adjacent microchannels during microchannel network formation. A. The fluorescence micro-architecture of single microchannels and numerous microchannels generated by breast epithelial cells (MCF-10A) and breast cancer cells (T47D, MCF-7, MDA-MB-231). Arrowheads represent microchannel connections. B. Western blot of MMP9, MMP2, and MMP14 across the four cell lines. C. Quantification of MMP14 protein levels normalized to GAPDH. D. Western blot of MMP9, MMP2, and MMP14 in MDA-MB-231 3D cultures at D_3, D_7, and D_{10}. E. Quantification of MMP14 expression level over time. F. Time-lapse confocal images (h:min) showing formation of a collagen bridge between two channels in live MDA-MB-231 cultures; arrowheads mark the emerging connection. Cell membrane (red), nuclei (blue), FITC–collagen (green). Unpaired two-tailed t-test. *P <0.05, ***P < 0.001. ****P < 0.0001. Scale bar is 20 μm.

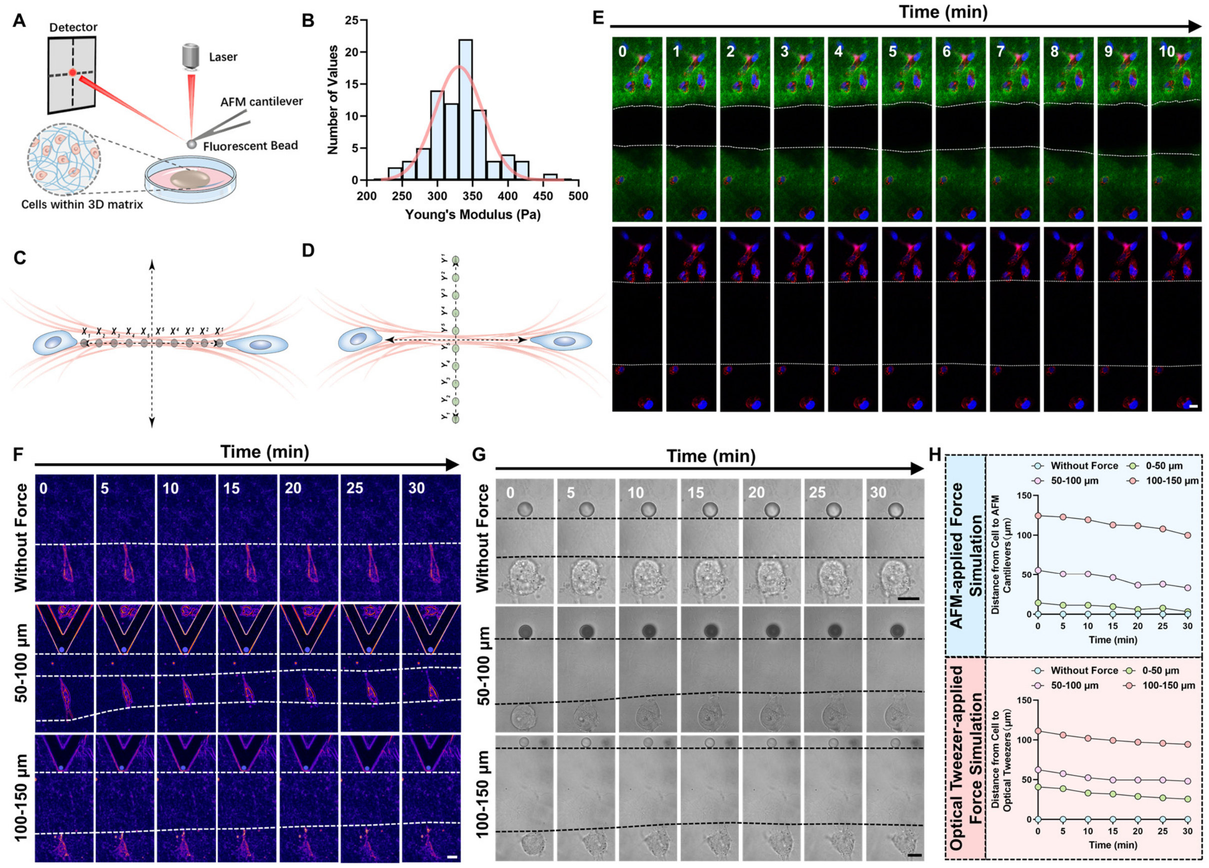

Fig. S4. Mechanical signals between bridged microchannels guide direct cell migration. A. AFM setup for measurement of the Young's moduli during live-cell within 3D matrix, the diameter of the fluorescent beads is 12 μm. B. Young's moduli distribution of the 3D collagen gels. C. AFM for measurement of Young's moduli during live-cell within 3D matrix. The marked points $X_{1\text{-}5}$ and $X^{1\text{-}5}$ show the locations of force-distance curves acquisition, which are symmetrically distributed, and the Young's modulus was calculated per 5 × 5 μm around the probing points. D. AFM for measurement of Young's moduli during live-cell within 3D matrix. The marked points $Y_{1\text{-}5}$ and $Y^{1\text{-}5}$ show the locations of force-distance curves acquisition, which is symmetrically distributed, and the Young's modulus was calculated per 5 × 5 μm around the probing points. E. Microdissection of a bridge revealed tensile loading: after cutting (time 0), the gap widened over time (white dashed outline). Cell membrane, nuclei, and FITC-collagen are shown in red, blue, and green, respectively. F. Time-lapse of MDA-MB-231 migration without stimulation (top) or with AFM-based force applied via the bead at 50–100 μm or 100–150 μm from the cell (middle, bottom). Images acquired every 5 minutes. G. Time-lapse of migration without stimulation (top)

or with optical-tweezer–based tensile cues generated by sinusoidal displacement of a trapped bead at 50–100 μm or 100–150 μm (middle, bottom). Bright circles denote the trapped bead; images acquired every 2–5 minites. H. Quantification of guidance: end-point distance between cells and the force application site for AFM-based and optical-tweezer–based assays at the indicated working distances. Scale bar is 20 μm.

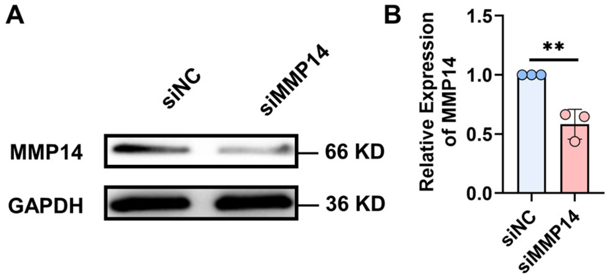

Fig. S5. Knockdown efficiency of siRNA targeting MMP14 and cell traction force. A-B. Relative protein expression of MMP14 in MDA-MB-231 cells transfected with siMMP14 or siNC. Western blots represent 3 independent experiments. Unpaired two-tailed t-test. **P <0.01.

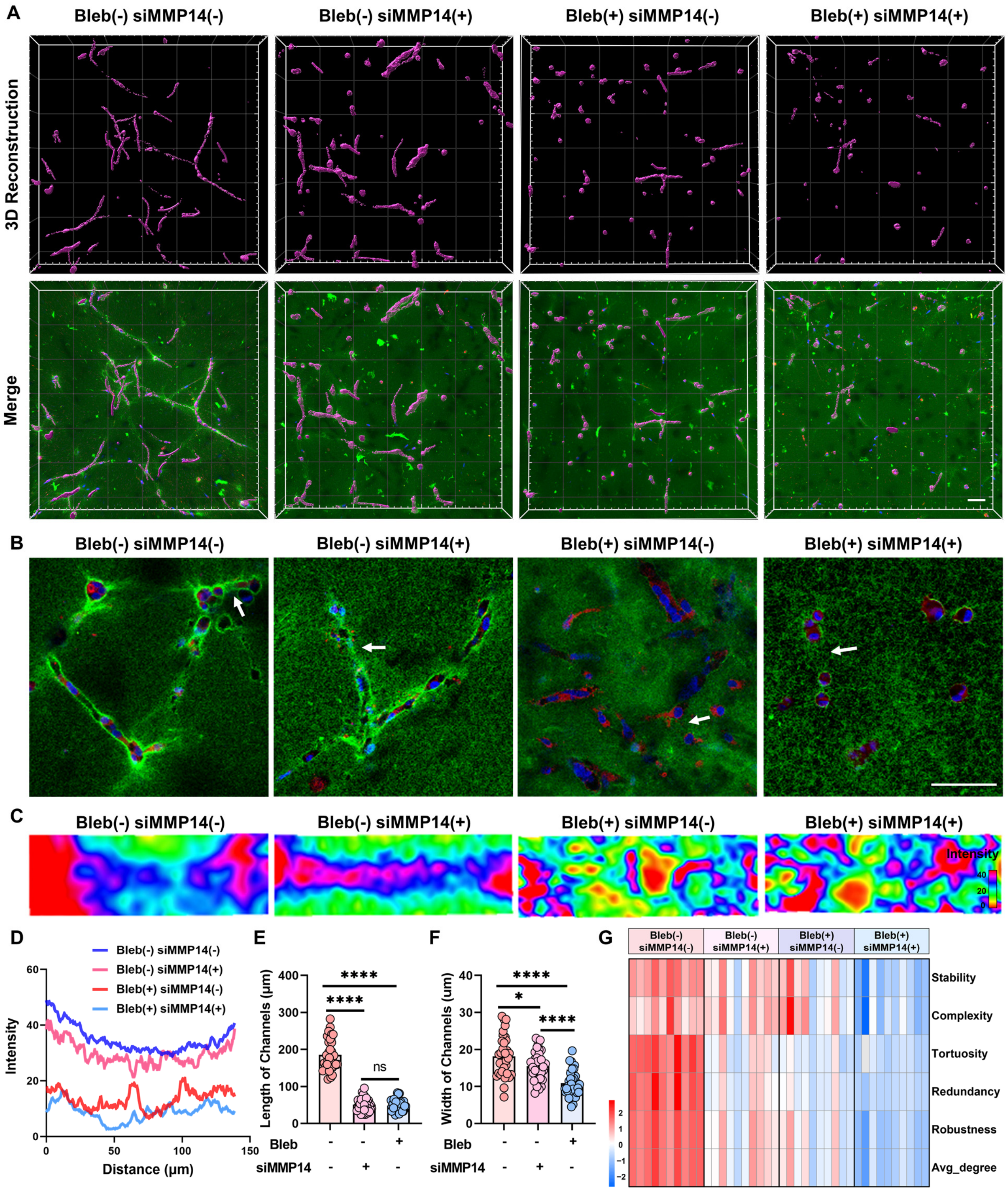

Fig. S6. Contractility is required for collagen-bridge formation to construct microchannel network. MDA-MB-231 cells were transfected with siMMP14 or siNC, treated ± blebbistatin (Bleb), and maintained in 3D culture for 7 days. A. 3D Imaris reconstructions (magenta) and merges with FITC–collagen (green), nuclei (blue) and membrane (red) of MDA-MB-231 cultures

at day 7 under four conditions: control, siMMP14, blebbistatin (Bleb), and Bleb + siMMP14. Both perturbations reduce channel formation; the combined treatment nearly abolishes it. B. Typical micro-architecture of microchannels at day 7 culture within 3D matrix. Cell plasma membrane (red), cell nucleus (blue), FITC-collagen matrix (green). Arrowheads represent microchannel connections. C. Pseudocolor collagen-intensity maps from the bridged regions in (B). D. Quantification of the local fluorescence intensity of the arrowheads marked regions among the four groups. E, F. Quantification of microchannel length (E) and width (F) per field for control, siMMP14, and Bleb groups (the Bleb + siMMP14 group was not analyzed due to near-absence of channels). G. Heatmap of network features (stability, complexity, tortuosity, redundancy, robustness, average degree) showing broad reductions with siMMP14 and/or Bleb. Data are presented as mean ± SD, bubbles represent individual fields. Unpaired two-tailed t-test. ns, not significant, $^*P < 0.05$, $^{****}P < 0.0001$. Scale bar is 50 μm.

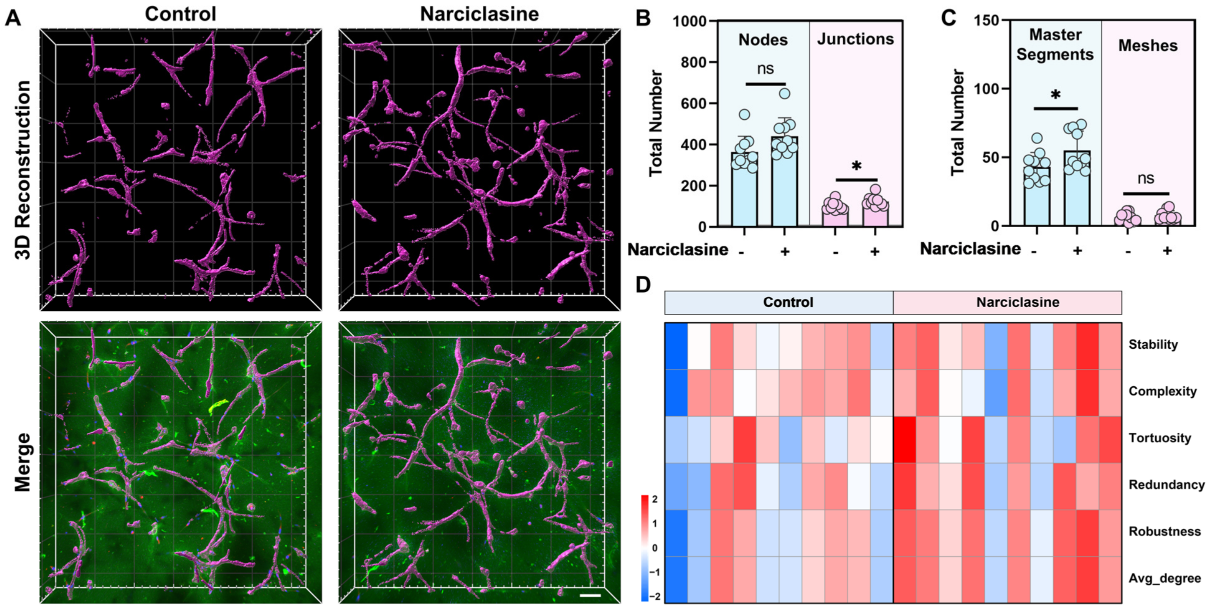

Fig. S7. Enhancement of contractility modestly promotes microchannel network assembly. A. 3D reconstructions of lumenized channels (magenta) and merges with FITC–collagen (green) and nuclei (blue) for MDA-MB-231 cultures with or without narciclasine (day 7). B. Quantification shows a reduction in junctions with control versus an increase with narciclasine, and a significant rise in master segments, whereas nodes and meshes are not significantly changed. D. Heatmap of network metrics (stability, complexity, tortuosity, redundancy, robustness, average degree) indicating an overall upward trend after narciclasine. Data are mean ± SD. points represent individual fields. Unpaired two-tailed t-test. ns, not significant; *$P < 0.05$. Scale bar, 50 μm.

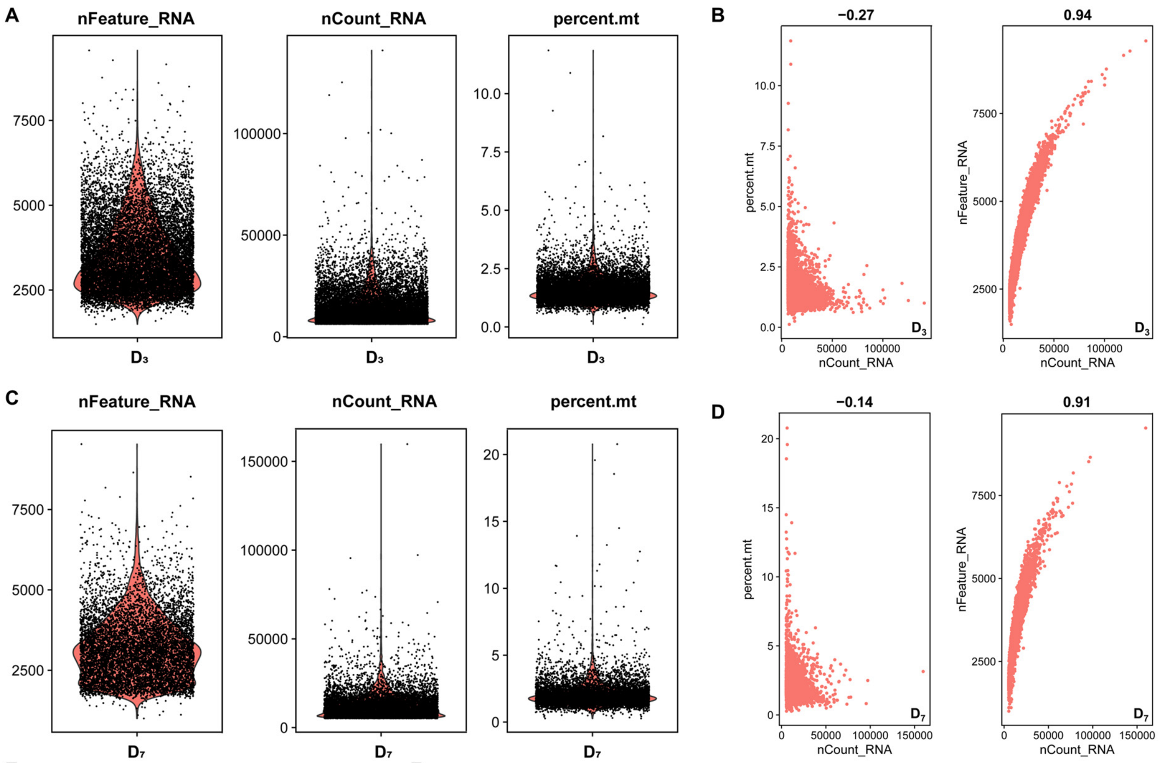

E

	D_3		D_7	
Cluster 0	4485	34.56%	3668	48.09%
Cluster 1	3956	30.55%	2032	26.64%
Cluster 2	3116	24.01%	1053	13.81%
Cluster 3	923	7.11%	706	9.26%
Cluster 4	432	3.33%	99	1.30%
Cluster 5	56	0.43%	69	0.90%
SUM	12,968		7,627	

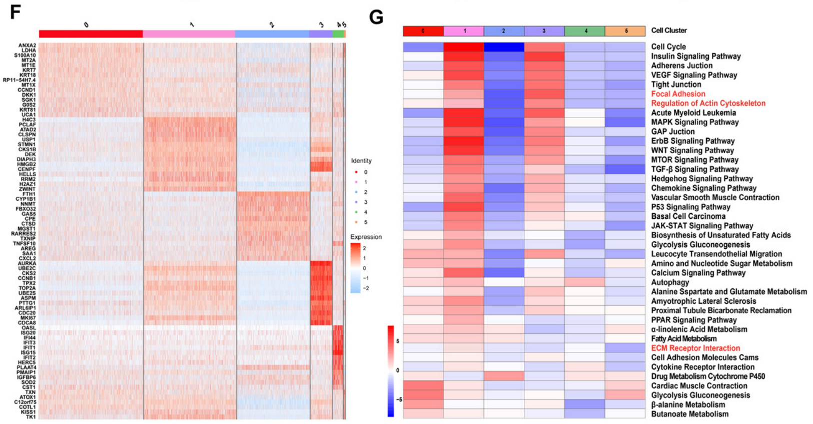

Fig. S8. Quality control for single-cell RNA sequencing, expression signatures and GSVA pathways of cell clusters at D_3. A. Violin/boxplots of number of genes (nFeature), number of counts (nCount), and the percentage of counts from mitochondrial genes (percent.mt) per cell sample at D_3. B. Correlation scatter plot of percent.mt and nCount across cells at at D_3. C. Violin/boxplots of number of genes (nFeature), number of counts (nCount), and the percentage of counts from mitochondrial genes (percent.mt) per cell sample at D_7. D. Correlation scatter plot of percent.mt and nCount across cells at at D_7. E. Table of the cell numbers in each cluster with percentage on total cells counted. F. Heatmap of top 15 differentially expressed genes (DEGs) of

the 6 clusters identified at D_3. G. Pathway activity of DEGs assessed by Gene Set Variation Analysis (GSVA) in each cell cluster at D_3.

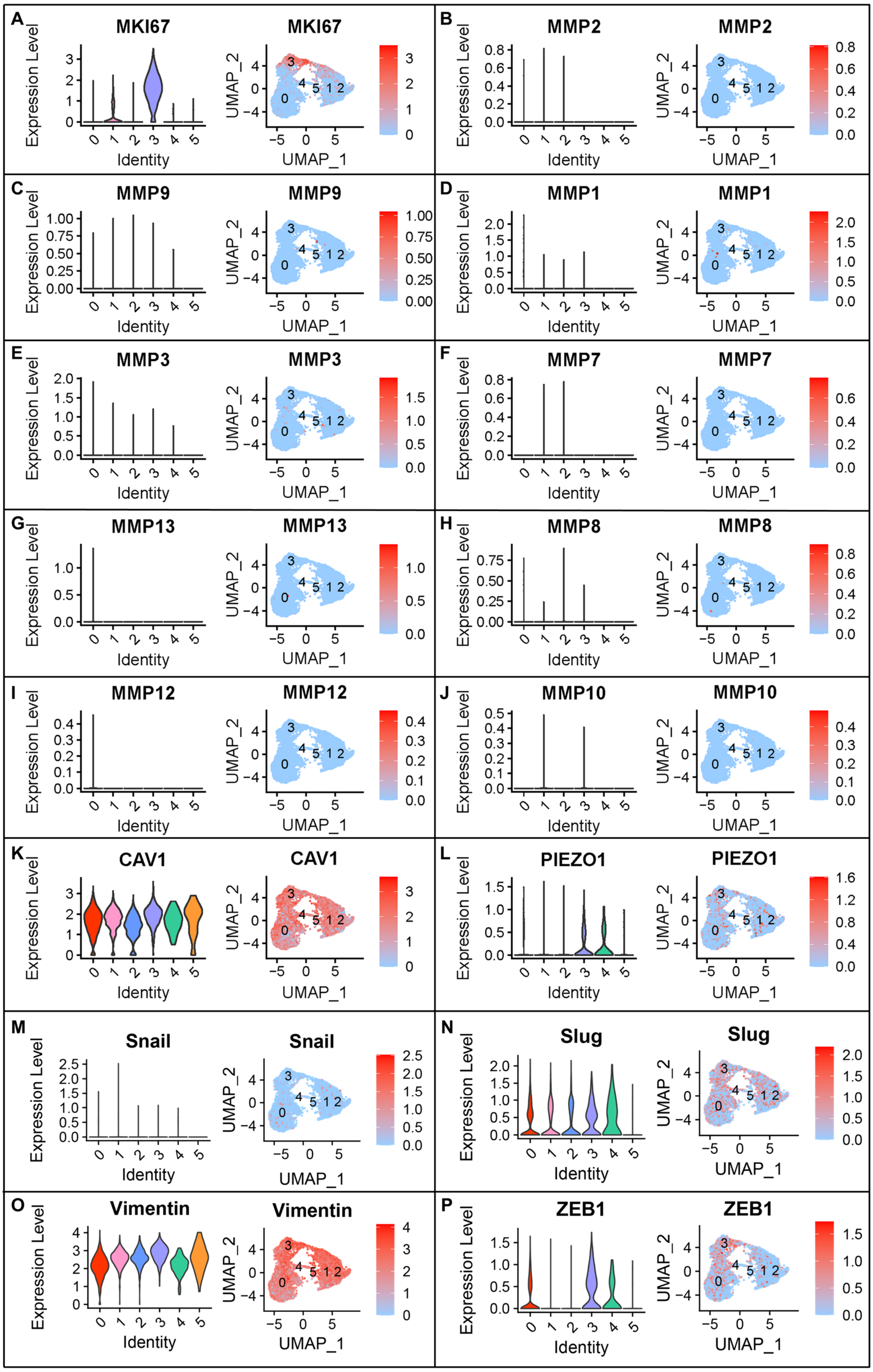

A
MKI67
B
MMP2
C
MMP9
D
MMP1
E
MMP3
F
MMP7
G
MMP13
H
MMP8
I
MMP12
J
MMP10
K
CAV1
L
PIEZO1
M
Snail
N
Slug
O
Vimentin
P
ZEB1
Expression Level
Identity
UMAP_1
UMAP_2

Fig. S9. Violin plots and UMAP visualizations of related genes from the 6 clusters at D_7. A. Expression level of MIKI67 (ki-67), which represent the invasiveness of cancer cells, indicating cluster 3 is the most invasive cell cluster. B-J. Expression levels of matrix MMPs involved in ECM degradation, specifically MMP2, MMP9, MMP1, MMP3, MMP7, MMP13, MMP8, MMP12, and MMP10, across the clusters. These MMPs are key mediators in tissue remodeling and metastasis. K, L. Expression level of the mechano-sensitive genes, CAV1 (L) and PIEZO1 (L), which play crucial roles in sensing mechanical cues and modulating cellular responses to physical forces in the microenvironment. M-P. Expression level of the Snail, Slug, Vimentin, and ZEB1.

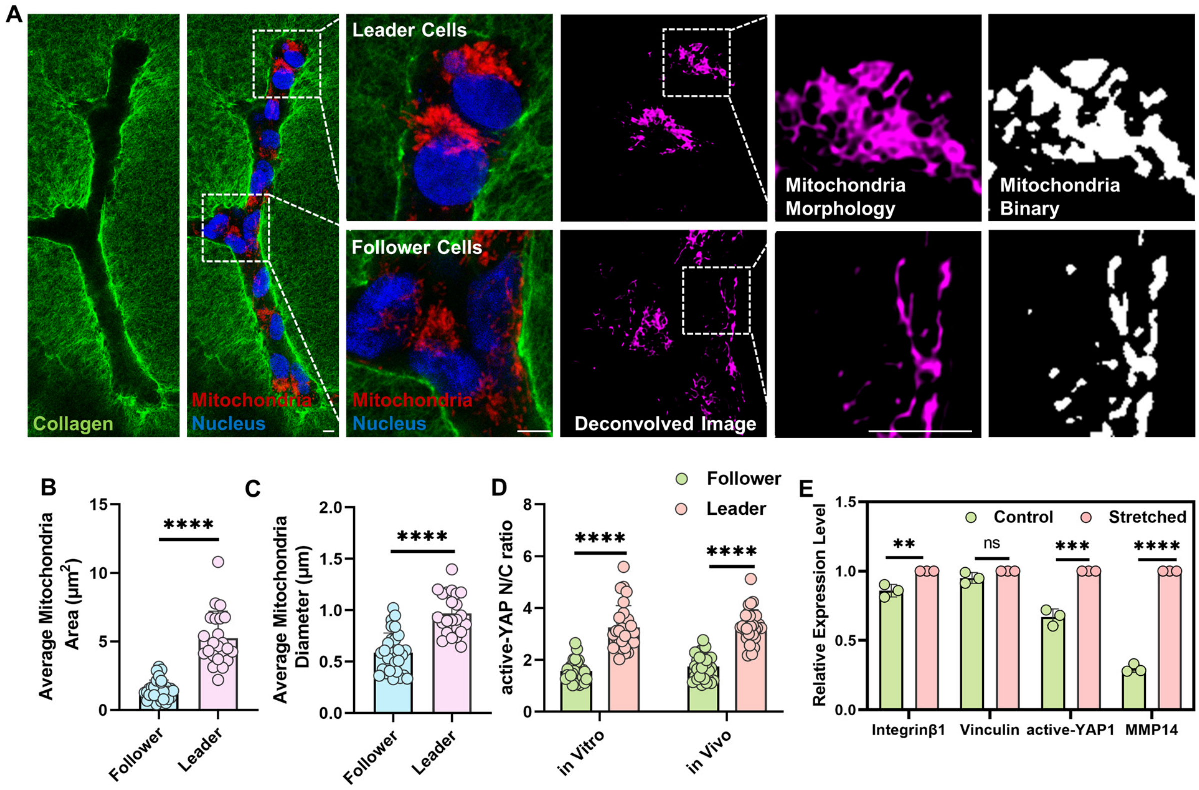

Fig. S10. Leader cells show higher mitochondrial activity and activated mechanotransduction. A. Confocal images of single microchannels in 3D FITC-collagen (green). Mitochondria (MitoTracker, red) and nuclei (DAPI, blue). Insets show representative leader and follower cells. At right are the corresponding deconvolved images and binarized masks used to quantify mitochondrial morphology. B-C. Quantification of mean mitochondrial area (B) and equivalent diameter (C) per cell. D. Active-YAP nuclear-to-cytoplasmic ratio for follower versus leader cells measured *in vitro* and *in vivo*. E. Relative protein expression in stretched versus control matrices for integrinβ1, vinculin, active-YAP, and MMP14. Data are mean ± SD. points represent individual fields. Unpaired two-tailed t-test. $^{**}P < 0.01$, $^{***}P < 0.001$, $^{****}P < 0.0001$, ns, not significant. Scale bar is 5 μm.

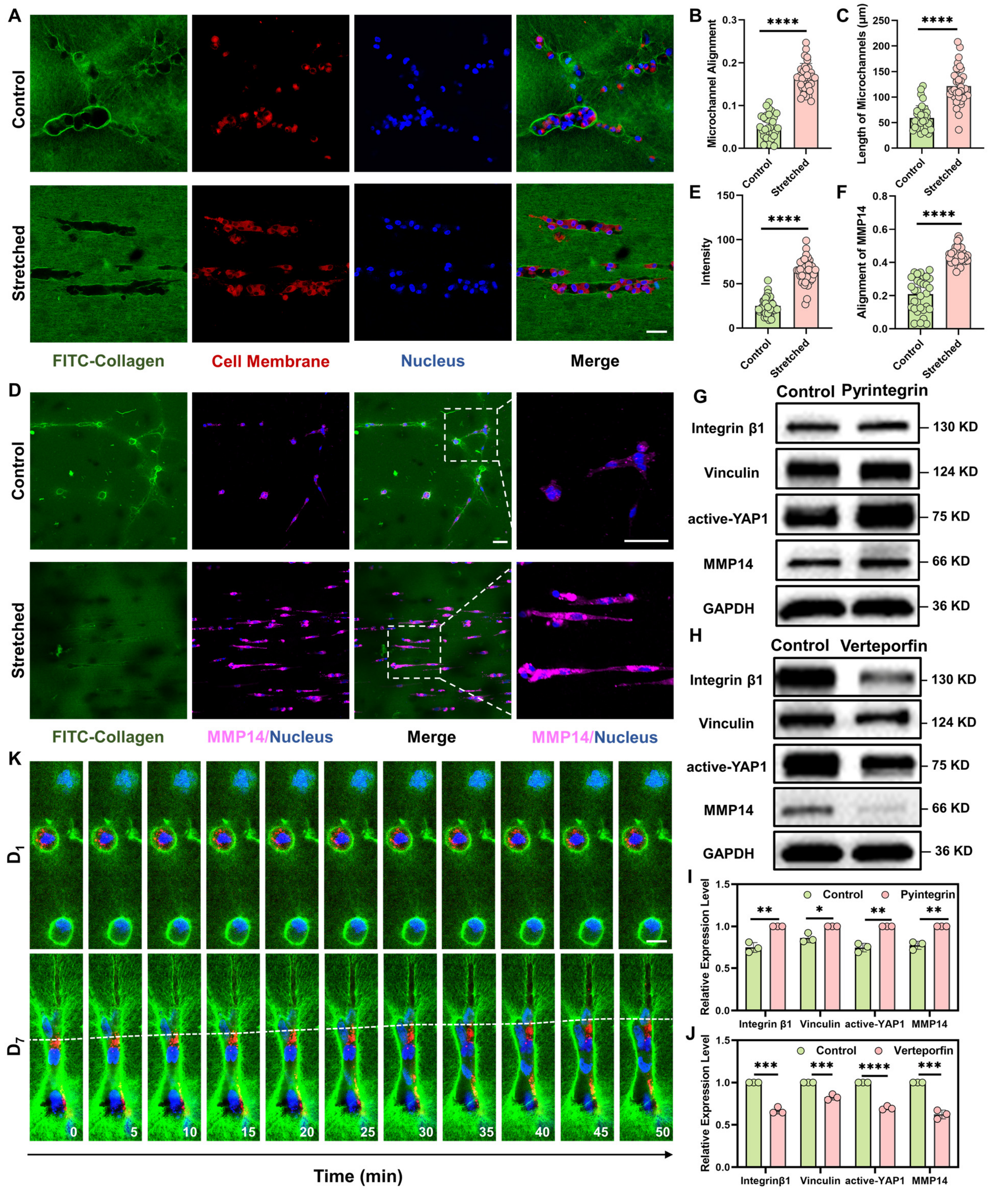

Fig. S11. Leader cells are enriched for MMP14 and mechanotransduction, and external stretch aligns microchannels. A. Representative 3D views of single microchannels formed for 3 days in random (Control) or uniaxially Stretched collagen. Channels align with the fiber axis under

stretch. FITC-collagen (green), cell membrane (red), nuclei (blue). B-C. Quantification of channel orientation (alignment to the stretch axis) (B) and channel length (C) on day 3. D. Immunofluorescence for MMP14 in Control versus Stretched gels showing enriched, elongated MMP14 signal along channel walls under stretch; zoom highlights accumulation at the front. E-F. Quantification of MMP14 signal under stretch: mean fluorescence intensity (E) and alignment index along channels (F) relative to Control. G-H. Western blots for integrinβ1, vinculin, active-YAP, and MMP14 in cells treated with the RhoA activator pyrintegrin (G) or the RhoA/YAP inhibitor verteporfin (H). I-J. Quantification of G–H normalized to GAPDH. K. Time-lapse frames (5-min intervals) of mixed populations in 3D collagen showing cells treated by RhoA activator pyrintegrin preferentially advancing at microchannel fronts. Data are mean ± SD; points represent individual fields. Unpaired two-tailed t-test. *$P < 0.05$, **$P < 0.01$, ***$P < 0.001$, ****$P < 0.0001$.

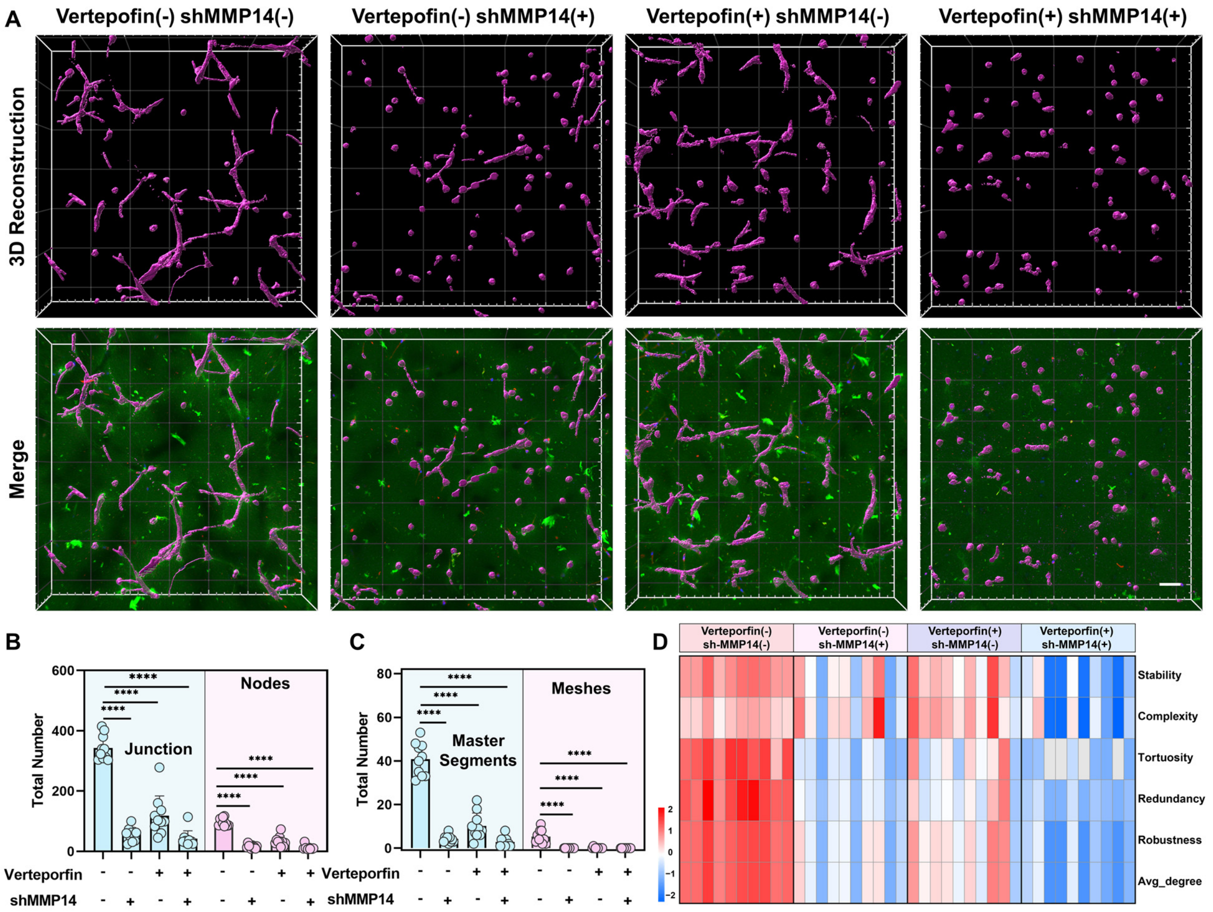

Fig. S12. Mechanotransduction and MMP14 intervention regulate microchannel network assembly. A. 3D reconstructions (magenta) and merges with FITC-collagen (green) of day-7 cultures under four conditions: Control, shMMP14 knockdown, verteporfin treatment, and combined verteporfin + shMMP14. B-C. Quantification of junctions and nodes per field (B) and of master segments and meshes (C). D. Heat map of network topology features (stability, complexity, tortuosity, redundancy, robustness, average degree) across the four conditions. Data are mean ± SD; points represent individual fields. Unpaired two-tailed t-test. ****$P < 0.0001$. Scale bars, 50 μm.

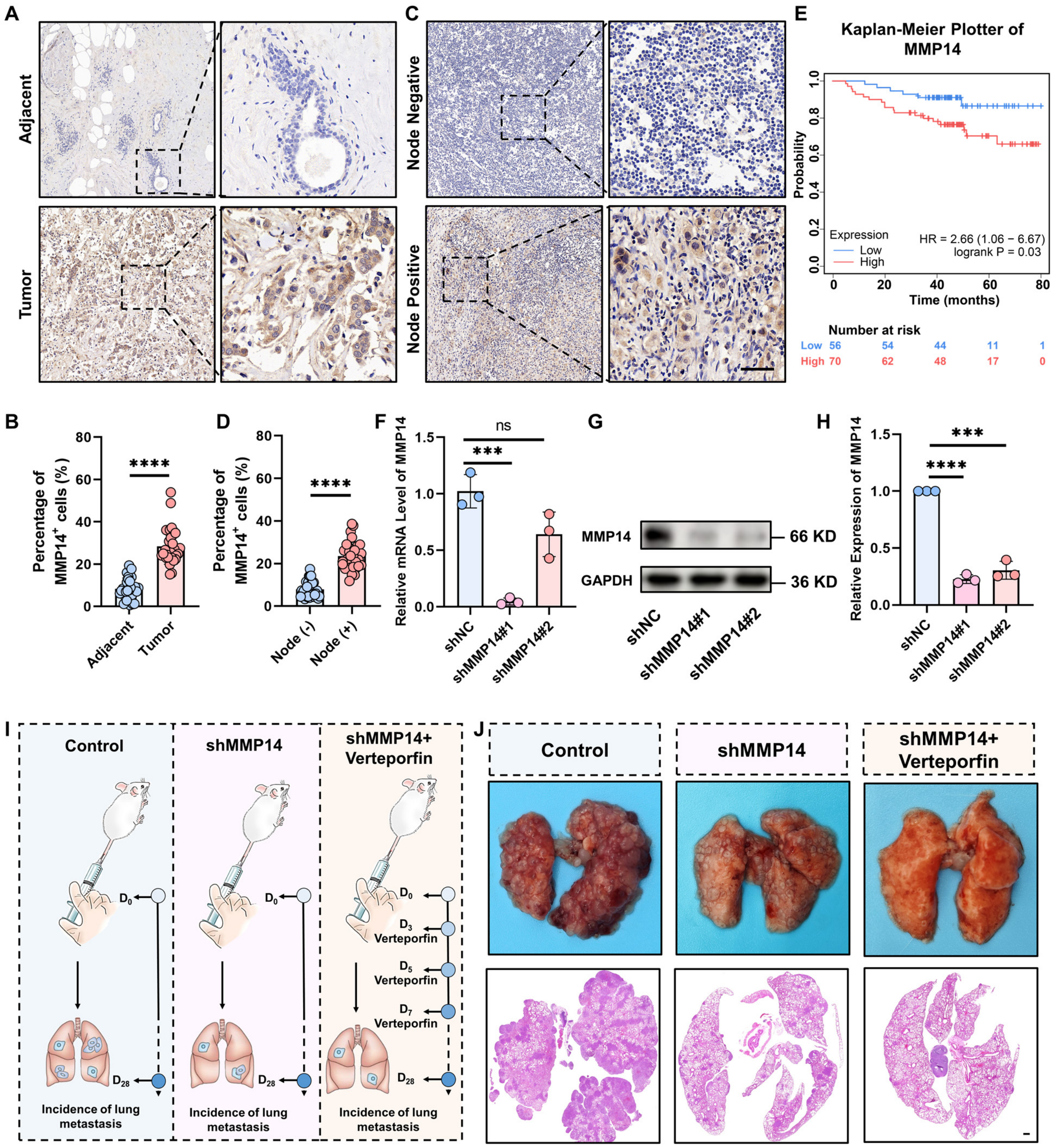

Fig. S13. Clinical, molecular, and *in vivo* evidence linking MMP14 to metastasis and its cooperation with mechanotransduction. A. Representative IHC for MMP14 in adjacent non-tumor breast tissue and matched tumors (left: low magnification; right: zoomed regions). B. Quantification of MMP14⁺ cells in adjacent versus tumor tissues. C. Representative IHC for MMP14 in node negative and node positive tumors. D. Quantification of MMP14⁺ cells in node negative versus node positive cases. E. Kaplan–Meier analysis (KM-plotter) showing poorer

survival in patients with high MMP14 expression in a cohort of triple negative breast cancer (https://kmplot.com), hazard ratio (HR) and log-rank *P* are indicated. F-H. Validation of MMP14 knockdown in MDA-MB-231 cells using two shRNAs: RT-qPCR (F), representative western blots (G), and quantification normalized to GAPDH (H). I. Schematic of the experimental design for the tail-vein lung metastasis model: control (shNC), shMMP14, and shMMP14 plus verteporfin. Verteporfin (100 mg/kg) was utilized on days 3, 5, and 7 after injection. J. Representative gross lungs and H&E sections at day 28 showing reduced metastatic burden with shMMP14 and a further decrease with the combination treatment. Data are mean ± SD; points represent individual fields. Unpaired two-tailed t-test. ***$P < 0.001$, ****$P < 0.0001$, ns, not significant. Scale bars, 50 μm.

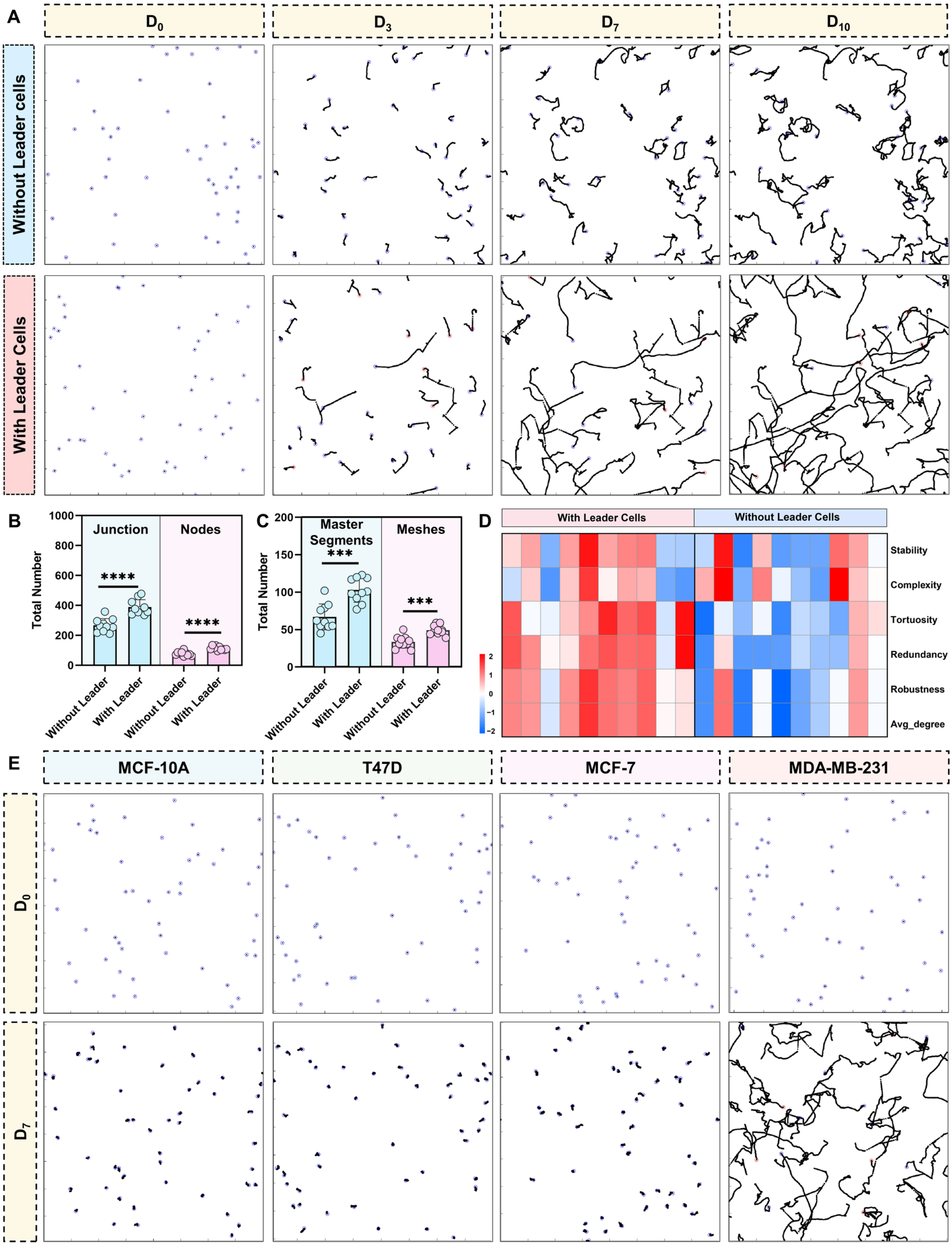

Fig. S14. Agent-based simulations show that leader cells accelerate network assembly and recapitulate cell-type differences. A. Time series of off-lattice simulations (D_0, D_3, D_7, D_{10}) run

without or with a leader subpopulation (endowed with higher traction and effective proteolysis). Leaders drive rapid formation of junction-rich, mesh-containing networks, whereas leader-free simulations yield only short, scattered tracks. B-C. Quantification of key network features showing significantly higher numbers of junctions, nodes, master segments, and mesh structures in simulations with leader cells. D. Heatmap comparing derived topological features between systems with and without leader cells, including metrics of network stability, complexity, tortuosity, redundancy, robustness, and average degree. E. Off-lattice simulations of ECM remodeling at D_0 and D_{10} for MCF-10A, T47D, MCF-7, and MDA-MB-231.

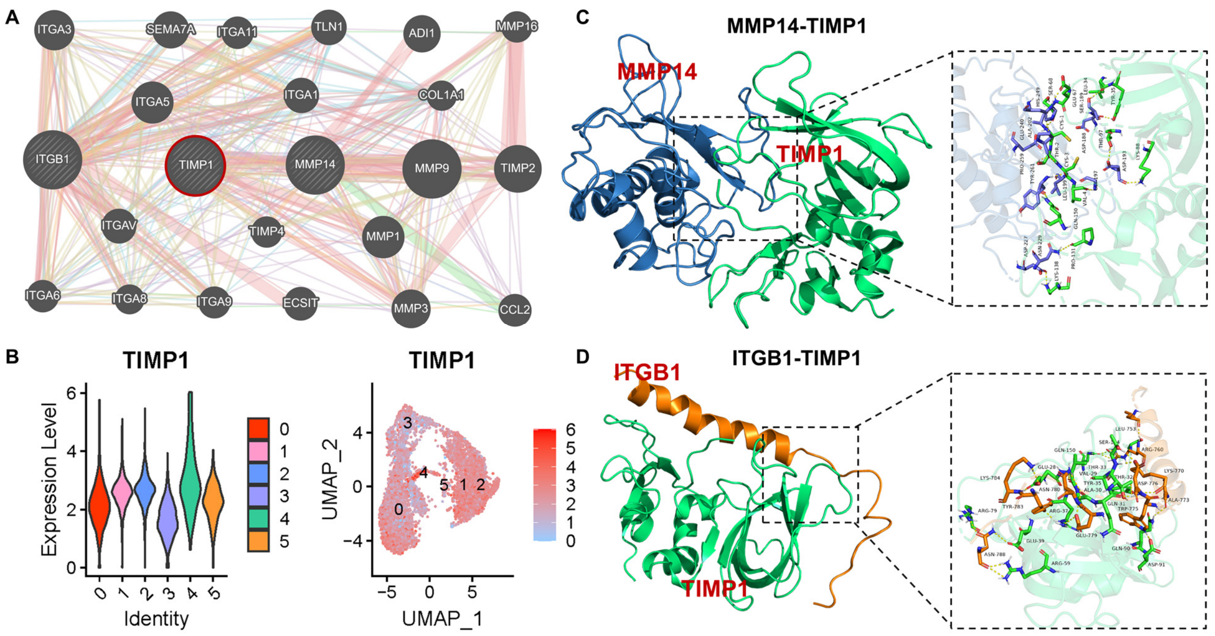

Fig. S15. TIMP1 as a potential modulator of crosstalk between MMP14 and ITGB1. A. Network visualization of protein-protein interactions among ECM-related and mechanotransduction molecules. TIMP1, outlined in red, occupies a central position within the network, with interactions involving key mechanotransduction proteins (e.g., ITGB1, ITGA3, and ITGA5) and ECM-degrading enzymes (e.g., MMP14, MMP1, and MMP9). B. Violin plot showing expression levels of TIMP1 across the clusters. C. 3D structural model displaying the interaction interface between MMP14 (blue) and TIMP1 (green). The magnified inset highlights specific amino acid residues that contribute to the stability of the protein interface, suggesting direct molecular interactions that may impact ECM remodeling processes. D. 3D structural model illustrating the interaction between ITGB1 (orange) and TIMP1 (green). The close-up view emphasizes critical amino acid interactions, reinforcing TIMP1's potential function in modulating the crosstalk between MMP14 and ITGB1.

Movie S1.

Microchannels are constructed by MDA-MB-231 cells on the 3rd day of culture.

Movie S2.

Numerous microchannels are widely interconnected when cultured for 7 days.

Movie S3.

Microchannel networks across the ECM are constructed when cultured for 10 days.

Movie S4.

Cells are confined in the 3D matrix. Scale bar is 100 μm.

Movie S5.

Microchannel network construction initiates unimpeded cancer cell metastasis. Scale bar is 100 μm.

Movie S6.

Mechanical bridge directs channel convergence. Scale bar is 50 μm.

Movie S7.

The dynamic construction of mechanical bridges. Scale bar is 50 μm.

Movie S8.

Off-lattice agent-based model of microchannel network formation in the presence of leader cells. Scale bar is 200 μm.

Movie S9.

Off-lattice agent-based model of microchannel network formation in the absence of leader cells. Scale bar is 200 μm.

Movie S10.

Off-lattice agent-based simulation of ECM remodeling by MCF-10A cells. No microchannel network is formed. Scale bar is 200 μm.

Movie S11.

Off-lattice agent-based simulation of ECM remodeling by T47D cells. No microchannel network is formed. Scale bar is 200 µm.

Movie S12.

Off-lattice agent-based simulation of ECM remodeling by MCF-7 cells. No microchannel network is formed. Scale bar is 200 µm.

Table S3. (separate file)

Table S3. Marker genes at D7.

Table S4. (separate file)

Table S4. Marker genes at D3.

Table S5. (separate file)

Table S5. Cell interaction pathways.

Table S6. Key Resource Table

Reagent or Resource	Source	Identifier
Antibodies		
Rabbit monoclonal to MMP-14	Abcam	Cat#ab51074
Rabbit monoclonal to MMP-9	Cell Signaling Technology	Cat#13667
Mouse monoclonal to MMP-2	Abcam	Cat#ab86607
Rabbit monoclonal to active YAP1	Abcam	Cat#ab205270
Rabbit monoclonal to Integrin beta 1	Abcam	Cat#ab52971
Rabbit monoclonal to Vinculin	Abcam	Cat#ab129002
anti-GAPDH antibody	Cell Signaling Technology	Cat#2118
Anti-rabbit IgG, HRP-linked Antibody	Cell Signaling Technology	Cat#7074
Goat anti-Rabbit IgG (H+L) Cross-Adsorbed Secondary Antibody, Alexa Fluor™ 647	Invitrogen	Cat#A-21244
Critical Commercial Assays		
Modified Masson's Trichrome Stain Kit	Solarbio	Cat#G1346
Omni-Easy™Instant BCA Protein Assay Kit	Epizyme	Cat#ZJ102
Chemicals, Peptides, and Recombinant Proteins		
Fluorescein Isothiocyanate	Yeasen	Cat#60514ES60
collagenase IV	Yeasen	Cat# 40510ES60
Triton-X100	Sigma-Aldrich	Cat#9036-19-5
Monodisperse fluorescent microspheres(Polystyrene microspheres)	Aladdin	Cat#M197276-5ml
Monodisperse fluorescent microspheres(Polystyrene microspheres)	Aladdin	Cat#M197277-5ml

Reagent or Resource	Source	Identifier
Green Fluorescent Microsphere	Dae	Cat#FL-PS-G-12
Verteporfin	MCE	Cat#HY-B0146
Pyrintegrin	MCE	Cat#HY-13306
Blebbistatin	Targetmol	Cat#T21550
Narciclasine	MCE	Cat# HY-16563
Lipofectamine 2000 reagent	Invitrogen	Cat#11668027
PDMS	Dow Corning	Cat#DC-184
Software and Algorithms		
ImageJ	NIH	https://imagej.nih.gov/ij/
Graphpad Prism 8	Graphpad	https://www.graphpad.com/scientific-software/prism/
Origin	OriginLab	https://www.originlab.com/
Imaris 10.1.0	Oxford Instruments (Imaris)	https://imaris.oxinst.com/
R 4.2.2	R	https://cran.r-project.org/mirrors.html
R studio	R	https://posit.co/download/rstudio-desktop/
Python	Python Software Foundation	https://www.python.org/
Matlab	The MathWorks, Inc.	https://www.mathworks.com/products/matlab.html
Other		
4′, 6-diamidino-2-phenylindole	Sigma-Aldrich	Cat#28718-90-3
NucBlue™ Live ReadyProbes™	Invitrogen	Cat#R37605

Reagent or Resource	Source	Identifier
Reagent (Hoechst 33342)		
CellMask^{TM} Orange Plasma Membrane Stain	Invitrogen	Cat#C10045
Mito-Tracker Red CMXRos	Beyotime	Cat#C1035-50μg
Non-Conductive Silicon Nitride	Bruker	Cat#NP-O10

REFERENCES

1. R. L. Siegel, T. B. Kratzer, A. N. Giaquinto, H. Sung, A. Jemal, Cancer statistics, 2025. *CA Cancer J. Clin.* **75**, 10–45 (2025).

2. K. M. Yamada, A. D. Doyle, J. Lu, Cell-3D matrix interactions: Recent advances and opportunities. *Trends Cell Biol.* **32**, 883–895 (2022).

3. D. Hoshino, K. C. Kirkbride, K. Costello, E. S. Clark, S. Sinha, N. Grega-Larson, M. J. Tyska, A. M. Weaver, Exosome secretion is enhanced by invadopodia and drives invasive behavior. *Cell Rep.* **5**, 1159–1168 (2013).

4. M. K. Driscoll, E. S. Welf, A. Weems, E. Sapoznik, F. Zhou, V. S. Murali, J. M. García-Arcos, M. Roh-Johnson, M. Piel, K. M. Dean, R. Fiolka, G. Danuser, Proteolysis-free amoeboid migration of melanoma cells through crowded environments via bleb-driven worrying. *Dev. Cell* **59**, 2414–2428.e8 (2024).

5. P. Friedl, K. Wolf, Tube travel: The role of proteases in individual and collective cancer cell invasion. *Cancer Res.* **68**, 7247–7249 (2008).

6. K. M. Wisdom, K. Adebowale, J. Chang, J. Y. Lee, S. Nam, R. Desai, N. S. Rossen, M. Rafat, R. B. West, L. Hodgson, O. Chaudhuri, Matrix mechanical plasticity regulates cancer cell migration through confining microenvironments. *Nat. Commun.* **9**, 4144 (2018).

7. P. Friedl, D. Gilmour, Collective cell migration in morphogenesis, regeneration and cancer. *Nat. Rev. Mol. Cell Biol.* **10**, 445–457 (2009).

8. P. Friedl, S. Alexander, Cancer invasion and the microenvironment: Plasticity and reciprocity. *Cell* **147**, 992–1009 (2011).

9. S. A. Vilchez Mercedes, F. Bocci, H. Levine, J. N. Onuchic, M. K. Jolly, P. K. Wong, Decoding leader cells in collective cancer invasion. *Nat. Rev. Cancer* **21**, 592–604 (2021).

10. M. R. Zanotelli, A. Rahman-Zaman, J. A. VanderBurgh, P. V. Taufalele, A. Jain, D. Erickson, F. Bordeleau, C. A. Reinhart-King, Energetic costs regulated by cell mechanics and

confinement are predictive of migration path during decision-making. *Nat. Commun.* **10**, 4185 (2019).

11. E. Armingol, A. Officer, O. Harismendy, N. E. Lewis, Deciphering cell–cell interactions and communication from gene expression. *Nat. Rev. Genet.* **22**, 71–88 (2021).

12. F. Alisafaei, D. Shakiba, Y. Hong, G. Ramahdita, Y. Huang, L. E. Iannucci, M. D. Davidson, M. Jafari, J. Qian, C. Qu, D. Ju, D. R. Flory, Y.-Y. Huang, P. Gupta, S. Jiang, A. Mujahid, S. Singamaneni, K. M. Pryse, P.-h. G. Chao, J. A. Burdick, S. P. Lake, E. L. Elson, N. Huebsch, V. B. Shenoy, G. M. Genin, Tension anisotropy drives fibroblast phenotypic transition by self-reinforcing cell–extracellular matrix mechanical feedback. *Nat. Mater.* **24**, 955–965 (2025).

13. O. Chaudhuri, S. T. Koshy, C. Branco da Cunha, J. W. Shin, C. S. Verbeke, K. H. Allison, D. J. Mooney, Extracellular matrix stiffness and composition jointly regulate the induction of malignant phenotypes in mammary epithelium. *Nat. Mater.* **13**, 970–978 (2014).

14. J. Chang, A. Saraswathibhatla, Z. Song, S. Varma, C. Sanchez, N. H. K. Alyafei, D. Indana, R. Slyman, S. Srivastava, K. Liu, M. C. Bassik, M. P. Marinkovich, L. Hodgson, V. Shenoy, R. B. West, O. Chaudhuri, Cell volume expansion and local contractility drive collective invasion of the basement membrane in breast cancer. *Nat. Mater.* **23**, 711–722 (2024).

15. K. Bera, A. Kiepas, I. Godet, Y. Li, P. Mehta, B. Ifemembi, C. D. Paul, A. Sen, S. A. Serra, K. Stoletov, J. Tao, G. Shatkin, S. J. Lee, Y. Zhang, A. Boen, P. Mistriotis, D. M. Gilkes, J. D. Lewis, C. M. Fan, A. P. Feinberg, M. A. Valverde, S. X. Sun, K. Konstantopoulos, Extracellular fluid viscosity enhances cell migration and cancer dissemination. *Nature* **611**, 365–373 (2022).

16.. Enríquez, S. Libring, T. C. Field, J. Jimenez, T. Lee, H. Park, D. Satoski, M. K. Wendt, S. Calve, A. B. Tepole, L. Solorio, H. Lee, High-throughput magnetic actuation platform for evaluating the effect of mechanical force on 3D tumor microenvironment. *Adv. Funct. Mater.* **31**, 2005021 (2021).

17. P. Kanchanawong, D. A. Calderwood, Organization, dynamics and mechanoregulation of integrin-mediated cell–ECM adhesions. *Nat. Rev. Mol. Cell Biol.* **24**, 142–161 (2023).

18. Y. Long, Y. Niu, K. Liang, Y. Du, Mechanical communication in fibrosis progression. *Trends Cell Biol.* **32**, 70–90 (2022).

19. Q. Shi, R. P. Ghosh, H. Engelke, C. H. Rycroft, L. Cassereau, J. A. Sethian, V. M. Weaver, J. T. Liphardt, Rapid disorganization of mechanically interacting systems of mammary acini. *Proc. Natl. Acad. Sci. U.S.A.* **111**, 658–663 (2014).

20. L. Liu, H. Yu, H. Zhao, Z. Wu, Y. Long, J. Zhang, X. Yan, Z. You, L. Zhou, T. Xia, Y. Shi, B. Xiao, Y. Wang, C. Huang, Y. Du, Matrix-transmitted paratensile signaling enables myofibroblast–fibroblast cross talk in fibrosis expansion. *Proc. Natl. Acad. Sci. U.S.A.* **117**, 10832–10838 (2020).

21. K. Wolf, P. Friedl, Extracellular matrix determinants of proteolytic and non-proteolytic cell migration. *Trends Cell Biol.* **21**, 736–744 (2011).

22. M. Tozluoğlu, A. L. Tournier, R. P. Jenkins, S. Hooper, P. A. Bates, E. Sahai, Matrix geometry determines optimal cancer cell migration strategy and modulates response to interventions. *Nat. Cell Biol.* **15**, 751–762 (2013).

23. R. Ferrari, G. Martin, O. Tagit, A. Guichard, A. Cambi, R. Voituriez, S. Vassilopoulos, P. Chavrier, MT1-MMP directs force-producing proteolytic contacts that drive tumor cell invasion. *Nat. Commun.* **10**, 4886 (2019).

24. A. Butler, P. Hoffman, P. Smibert, E. Papalexi, R. Satija, Integrating single-cell transcriptomic data across different conditions, technologies and species. *Nat. Biotechnol.* **36**, 411–420 (2018).

25. R. Satija, J. A. Farrell, D. Gennert, A. F. Schier, A. Regev, Spatial reconstruction of single-cell gene expression data. *Nat. Biotechnol.* **33**, 495–502 (2015).

26. B. Geiger, J. P. Spatz, A. D. Bershadsky, Environmental sensing through focal adhesions. *Nat. Rev. Mol. Cell Biol.* **10**, 21–33 (2009).

27. P. Kanchanawong, G. Shtengel, A. M. Pasapera, E. B. Ramko, M. W. Davidson, H. F. Hess, C. M. Waterman, Nanoscale architecture of integrin-based cell adhesions. *Nature* **468**, 580–584 (2010).

28. E. R. Horton, J. D. Humphries, J. James, M. C. Jones, J. A. Askari, M. J. Humphries, The integrin adhesome network at a glance. *J. Cell Sci.* **129**, 4159–4163 (2016).

29. A. Dongre, R. A. Weinberg, New insights into the mechanisms of epithelial–mesenchymal transition and implications for cancer. *Nat. Rev. Mol. Cell Biol.* **20**, 69–84 (2019).

30. X. Sun, B. Wu, H.-C. Chiang, H. Deng, X. Zhang, W. Xiong, J. Liu, A. M. Rozeboom, B. T. Harris, E. Blommaert, A. Gomez, R. E. Garcia, Y. Zhou, P. Mitra, M. Prevost, D. Zhang, D. Banik, C. Isaacs, D. Berry, C. Lai, K. Chaldekas, P. S. Latham, C. A. Brantner, A. Popratiloff, V. X. Jin, N. Zhang, Y. Hu, M. A. Pujana, T. J. Curiel, Z. An, R. Li, Tumour DDR1 promotes collagen fibre alignment to instigate immune exclusion. *Nature* **599**, 673–678 (2021).

31. K. H. Palmquist, S. F. Tiemann, F. L. Ezzeddine, S. Yang, C. R. Pfeifer, A. Erzberger, A. R. Rodrigues, A. E. Shyer, Reciprocal cell-ECM dynamics generate supracellular fluidity underlying spontaneous follicle patterning. *Cell* **185**, 1960–1973.e11 (2022).

32. C. M. Kraning-Rush, S. P. Carey, M. C. Lampi, C. A. Reinhart-King, Microfabricated collagen tracks facilitate single cell metastatic invasion in 3D. *Integr. Biol.* **5**, 606–616 (2013).

33. P. Blinder, P. S. Tsai, J. P. Kaufhold, P. M. Knutsen, H. Suhl, D. Kleinfeld, The cortical angiome: An interconnected vascular network with noncolumnar patterns of blood flow. *Nat. Neurosci.* **16**, 889–897 (2013).

34. Z. Zou, T. Tang, E. Macías-Sánchez, S. Sviben, W. J. Landis, L. Bertinetti, P. Fratzl, Three-dimensional structural interrelations between cells, extracellular matrix, and mineral in normally mineralizing avian leg tendon. *Proc. Natl. Acad. Sci. U.S.A.* **117**, 14102–14109 (2020).

35. O. Chepizhko, J.-M. Armengol-Collado, S. Alexander, E. Wagena, B. Weigelin, L. Giomi, P. Friedl, S. Zapperi, C. A. M. La Porta, Confined cell migration along extracellular matrix space in vivo. *Proc. Natl. Acad. Sci. U.S.A.* **122**, e2414009121 (2025).

36. Q. Fan, Y. Zheng, X. Wang, R. Xie, Y. Ding, B. Wang, X. Yu, Y. Lu, L. Liu, Y. Li, M. Li, Y. Zhao, Y. Jiao, F. Ye, Dynamically re-organized collagen fiber bundles transmit mechanical signals and induce strongly correlated cell migration and self-organization. *Angew. Chem. Int. Ed. Engl.* **60**, 11858–11867 (2021).

37. J. Kim, J. Feng, C. A. R. Jones, X. Mao, L. M. Sander, H. Levine, B. Sun, Stress-induced plasticity of dynamic collagen networks. *Nat. Commun.* **8**, 842 (2017).

38. A. G. Clark, A. Maitra, C. Jacques, M. Bergert, C. Pérez-González, A. Simon, L. Lederer, A. Diz-Muñoz, X. Trepat, R. Voituriez, D. M. Vignjevic, Self-generated gradients steer collective migration on viscoelastic collagen networks. *Nat. Mater.* **21**, 1200–1210 (2022).

39. K. Wolf, Y. I. Wu, Y. Liu, J. Geiger, E. Tam, C. Overall, M. S. Stack, P. Friedl, Multi-step pericellular proteolysis controls the transition from individual to collective cancer cell invasion. *Nat. Cell Biol.* **9**, 893–904 (2007).

40. C. Gaggioli, S. Hooper, C. Hidalgo-Carcedo, R. Grosse, J. F. Marshall, K. Harrington, E. Sahai, Fibroblast-led collective invasion of carcinoma cells with differing roles for RhoGTPases in leading and following cells. *Nat. Cell Biol.* **9**, 1392–1400 (2007).

41. C. L. Marchant, A. N. Malmi-Kakkada, J. A. Espina, E. H. Barriga, Cell clusters softening triggers collective cell migration in vivo. *Nat. Mater.* **21**, 1314–1323 (2022).

42. P. Friedl, M. Zegers, A collective strategy to promote the dissemination of single cancer cells. *J. Cell Biol.* **223**, e202405014 (2024).

43. B. Weigelin, G. J. Bakker, P. Friedl, Intravital third harmonic generation microscopy of collective melanoma cell invasion: Principles of interface guidance and microvesicle dynamics. *Intravital* **1**, 32–43 (2012).

44. A. Haeger, S. Alexander, M. Vullings, F. M. P. Kaiser, C. Veelken, U. Flucke, G. E. Koehl, M. Hirschberg, M. Flentje, R. M. Hoffman, E. K. Geissler, S. Kissler, P. Friedl, Collective cancer invasion forms an integrin-dependent radioresistant niche. *J. Exp. Med.* **217**, e20181184 (2019).

45. K. J. Cheung, E. Gabrielson, Z. Werb, A. J. Ewald, Collective invasion in breast cancer requires a conserved basal epithelial program. *Cell* **155**, 1639–1651 (2013).

46. W.-H. Jung, N. Yam, C.-C. Chen, K. Elawad, B. Hu, Y. Chen, Force-dependent extracellular matrix remodeling by early-stage cancer cells alters diffusion and induces carcinoma-associated fibroblasts. *Biomaterials* **234**, 119756 (2020).

47. O. Ilina, P. G. Gritsenko, S. Syga, J. Lippoldt, C. A. M. La Porta, O. Chepizhko, S. Grosser, M. Vullings, G. J. Bakker, J. Starruß, P. Bult, S. Zapperi, J. A. Käs, A. Deutsch, P. Friedl, Cell–cell adhesion and 3D matrix confinement determine jamming transitions in breast cancer invasion. *Nat. Cell Biol.* **22**, 1103–1115 (2020).

48. V. L. Cross, Y. Zheng, N. Won Choi, S. S. Verbridge, B. A. Sutermaster, L. J. Bonassar, C. Fischbach, A. D. Stroock, Dense type I collagen matrices that support cellular remodeling and microfabrication for studies of tumor angiogenesis and vasculogenesis in vitro. *Biomaterials* **31**, 8596–8607 (2010).

49. D. Pei, M. Wang, W. Li, M. Li, Q. Liu, R. Ding, J. Zhao, A. Li, J. Li, F. Xu, G. Jin, Remodeling of aligned fibrous extracellular matrix by encapsulated cells under mechanical stretching. *Acta Biomater.* **112**, 202–212 (2020).

50. J. P. Butler, I. M. Tolić-Nørrelykke, B. Fabry, J. J. Fredberg, Traction fields, moments and strain energy that cells exert on their surroundings. *Am. J. Physiol. Cell Physiol.* **282**, C595–C605 (2002).

51. O. Maiques, M. C. Sallan, R. Laddach, P. Pandya, A. Varela, E. Crosas-Molist, J. Barcelo, O. Courbot, Y. Liu, V. Graziani, Y. Arafat, J. Sewell, I. Rodriguez-Hernandez, B. Fanshawe, Y. Jung-Garcia, P. R. C. Imbert, E. M. Grasset, J. Albrengues, M. Santacana, A. Macià, J. Tarragona, X. Matias-Guiu, R. M. Marti, S. Tsoka, C. Gaggioli, J. L. Orgaz, G. O. Fruhwirth,

F. Wallberg, K. Betteridge, C. C. Reyes-Aldasoro, S. Haider, A. Braun, S. N. Karagiannis, A. Elosegui-Artola, V. Sanz-Moreno, Matrix mechano-sensing at the invasive front induces a cytoskeletal and transcriptional memory supporting metastasis. *Nat. Commun.* **16**, 1394 (2025).

52. A. L. Godeau, A. Seriola, O. Tchaicheeyan, M. Casals, D. Denkova, E. Aroca, O. Massafret, A. Parra, M. Demestre, A. Ferrer-Vaquer, S. Goren, A. Veiga, M. Solé, M. Boada, J. Comelles, E. Martínez, J. Colombelli, A. Lesman, S. Ojosnegros, Traction force and mechanosensitivity mediate species-specific implantation patterns in human and mouse embryos. *Sci. Adv.* **11**, eadr5199 (2025).

53. J. L. Killian, F. Ye, M. D. Wang, Optical tweezers: A force to be reckoned with. *Cell* **175**, 1445–1448 (2018).

54. T. S. Andrews, V. Y. Kiselev, D. McCarthy, M. Hemberg, Tutorial: Guidelines for the computational analysis of single-cell RNA sequencing data. *Nat. Protoc.* **16**, 1–9 (2021).

55. E. Becht, L. McInnes, J. Healy, C.-A. Dutertre, I. W. H. Kwok, L. G. Ng, F. Ginhoux, E. W. Newell, Dimensionality reduction for visualizing single-cell data using UMAP. *Nat. Biotechnol.* **37**, 38–44 (2019).

56. A. Liberzon, C. Birger, H. Thorvaldsdóttir, M. Ghandi, J. P. Mesirov, P. Tamayo, The Molecular Signatures Database (MSigDB) hallmark gene set collection. *Cell Syst.* **1**, 417–425 (2015).

57. S. Hänzelmann, R. Castelo, J. Guinney, GSVA: Gene set variation analysis for microarray and RNA-seq data. *BMC Bioinformatics* **14**, 7 (2013).

58. S. Jin, C. F. Guerrero-Juarez, L. Zhang, I. Chang, R. Ramos, C.-H. Kuan, P. Myung, M. V. Plikus, Q. Nie, Inference and analysis of cell-cell communication using CellChat. *Nat. Commun.* **12**, 1088 (2021).

59. M. Kanehisa, S. Goto, KEGG: Kyoto Encyclopedia of Genes and Genomes. *Nucleic Acids Res.* **28**, 27–30 (2000).

60. F. Lefranc, S. Sauvage, G. Van Goietsenoven, V. Mégalizzi, D. Lamoral-Theys, O. Debeir, S. Spiegl-Kreinecker, W. Berger, V. Mathieu, C. Decaestecker, R. Kiss, Narciclasine, a plant growth modulator, activates Rho and stress fibers in glioblastoma cells. *Mol. Cancer Ther.* **8**, 1739–1750 (2009).

61. A. Lánczky, B. Győrffy, Web-based survival analysis tool tailored for medical research (KMplot): Development and implementation. *J. Med. Internet Res.* **23**, e27633 (2021).

62. J. Schindelin, I. Arganda-Carreras, E. Frise, V. Kaynig, M. Longair, T. Pietzsch, S. Preibisch, C. Rueden, S. Saalfeld, B. Schmid, J.-Y. Tinevez, D. J. White, V. Hartenstein, K. Eliceiri, P. Tomancak, A. Cardona, Fiji: An open-source platform for biological-image analysis. *Nat. Methods* **9**, 676–682 (2012).

63. G. Carpentier, S. Berndt, S. Ferratge, W. Rasband, M. Cuendet, G. Uzan, P. Albanese, Angiogenesis analyzer for ImageJ: A comparative morphometric analysis of "endothelial tube formation Assay" and "Fibrin Bead Assay". *Sci. Rep.* **10**, 11568 (2020).

64. F. Blanchini, E. Franco, Structurally robust biological networks. *BMC Syst. Biol.* **5**, 74 (2011).

65. M. Aldana, P. Cluzel, A natural class of robust networks. *Proc. Natl. Acad. Sci. U.S.A.* **100**, 8710–8714 (2003).

66. I. Y. Wong, S. Javaid, E. A. Wong, S. Perk, D. A. Haber, M. Toner, D. Irimia, Collective and individual migration following the epithelial–mesenchymal transition. *Nat. Mater.* **13**, 1063–1071 (2014).

67. M. E. J. Newman, *Networks* (Oxford Univ. Press, 2010).

68. V. Latora, M. Marchiori, Efficient behavior of small-world networks. *Phys. Rev. Lett.* **87**, 198701 (2001).

69. E. Bullitt, G. Gerig, S. M. Pizer, W. Lin, S. R. Aylward, Measuring tortuosity of the intracerebral vasculature from MRA images. *IEEE Trans. Med. Imaging* **22**, 1163–1171 (2003).

70. R. Albert, H. Jeong, A.-L. Barabási, Error and attack tolerance of complex networks. *Nature* **406**, 378–382 (2000).
